# Targeting of insect epicuticular lipids by the entomopathogenic fungus *Beauveria bassiana*: hydrocarbon oxidation within the context of a host-pathogen interaction

**DOI:** 10.3389/fmicb.2013.00024

**Published:** 2013-02-15

**Authors:** Nicolás Pedrini, Almudena Ortiz-Urquiza, Carla Huarte-Bonnet, Shizhu Zhang, Nemat O. Keyhani

**Affiliations:** ^1^Facultad de Ciencias Médicas, Instituto de Investigaciones Bioquímicas de La Plata (CCT La Plata CONICET-UNLP)La Plata, Argentina; ^2^Department of Microbiology and Cell Science, University of FloridaGainesville, FL, USA; ^3^Jiangsu Key Laboratory for Microbes and Functional Genomics, Jiangsu Engineering and Technology Research Center for Microbiology, College of Life Sciences, Nanjing Normal UniversityNanjing, China

**Keywords:** *B. basiana*, entomopathogenic fungi, epicuticle, hydrocarbon degradation, cytochrome P450, host-pathogen coevolution

## Abstract

Broad host range entomopathogenic fungi such as *Beauveria bassiana* attack insect hosts via attachment to cuticular substrata and the production of enzymes for the degradation and penetration of insect cuticle. The outermost epicuticular layer consists of a complex mixture of non-polar lipids including hydrocarbons, fatty acids, and wax esters. Long chain hydrocarbons are major components of the outer waxy layer of diverse insect species, where they serve to protect against desiccation and microbial parasites, and as recognition molecules or as a platform for semiochemicals. Insect pathogenic fungi have evolved mechanisms for overcoming this barrier, likely with sets of lipid degrading enzymes with overlapping substrate specificities. Alkanes and fatty acids are substrates for a specific subset of fungal cytochrome P450 monooxygenases involved in insect hydrocarbon degradation. These enzymes activate alkanes by terminal oxidation to alcohols, which are further oxidized by alcohol and aldehyde dehydrogenases, whose products can enter β-oxidation pathways. *B. bassiana* contains at least 83 genes coding for cytochrome P450s (CYP), a subset of which are involved in hydrocarbon oxidation, and several of which represent new CYP subfamilies/families. Expression data indicated differential induction by alkanes and insect lipids and four CYP proteins have been partially characterized after heterologous expression in yeast. Gene knockouts revealed a phenotype for only one (*cyp52X1*) out of six genes examined to date. CYP52X1 oxidizes long chain fatty acids and participates in the degradation of specific epicuticular lipid components needed for breaching the insect waxy layer. Examining the hydrocarbon oxidizing CYP repertoire of pathogens involved in insect epicuticle degradation can lead to the characterization of enzymes with novel substrate specificities. Pathogen targeting may also represent an important co-evolutionary process regarding insect cuticular hydrocarbon synthesis.

## Introduction

Insect cuticles are a significant source of hydrocarbons in terrestrial ecosystems and remediation and turnover of these compounds is critical for the maintenance and flux of normal carbon cycles. Yeasts and filamentous fungi are known to degrade *n-alkanes* and although significant portions of the biochemical pathways regarding alkane catabolism have been described, much remains obscure. The insect epicuticle or waxy layer represents the first barrier to environmental threats including external compounds such as chemical and biological pesticides. This thin layer on the outer surface of the insect is comprised of a complex mixture of lipids that include abundant amounts of straight-chain and methyl-branched, saturated and unsaturated hydrocarbons.

**Figure 1:**
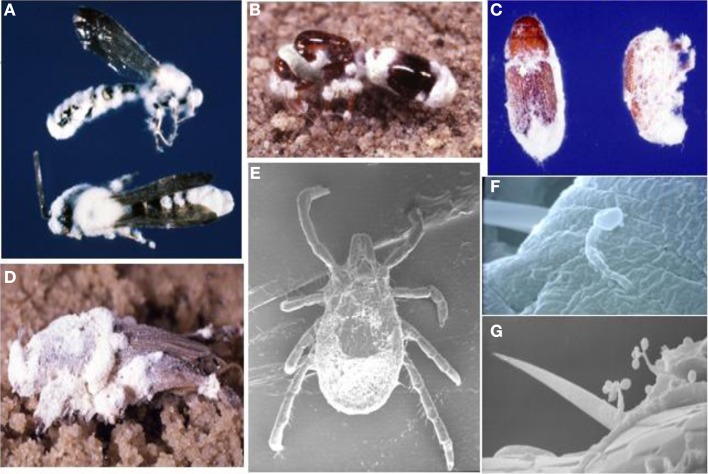
***B. bassiana* has an exceptionally broad host range that spans across Arthropoda classes, from insects including; wasps (A), fire ants (B), bark beetles (C), and mole crickets (D) to arachnids such as mites and ticks (E).** Cuticle penetration **(F)** and conidiogenesis from host cadaver **(G)** are also illustrated. (Images courtesy of D. Boucias and N. O. Keyhani).

Pathogenicity to invertebrates is represented by primitive fungi and is postulated to have arisen simultaneously with the emergence of insects approximately 500 million years ago (Berbee and Taylor, 2001). The ancient Chinese noted the lethal effects of fungi on silkworms and cicadas more than 2 millennia ago (Roberts and Humber, 1981) and Augustino Bassi in the 1830s used strains of *Beauveria (bassi)ana* as a model for his germ theory of disease in animals (Steinhaus, 1956). Due to their dispersal within most major fungal taxonomic groups, fungal-insect pathogens represent lifestyle adaptations that have likely evolved numerous times (Khachatourians, 1996; Goettel et al., 2000). *B. bassiana* has an exceptionally broad host range and is being studied for use as a biological control for a diverse range of insects (Figure 1). This host range includes insects that act as disease vectors and nuisance pests, crop pests, and even ecologically hazardous, invading pests, with recent studies highlighting the potential of entomopathogenic fungi as agents in combating the spread of malaria by controlling mosquito populations and in protecting agricultural crops from marauding locusts (Inglis et al., 2001; Kirkland et al., 2004b; Scholte et al., 2005; Fan et al., 2012a,b).

*B. bassiana* is a facultative saprophyte that belongs to the Hypocrealean order within the Ascomycota, and has evolved sophisticated mechanisms for penetrating the formidable barrier that constitutes the insect/arthropod exoskeleton or integument (Ferron, 1981; Binnington and Retnakaran, 1991; St Leger, 1991; Clarkson and Charnley, 1996). Interspersed within the cuticle barrier are biochemical components such as toxic lipids and phenols, enzyme inhibitors, proteins, and other defensive compounds that entomopathogens must overcome for successful virulence (Hackman, 1984; Renobales et al., 1991; Anderson et al., 1995). Pathogens must cope with hydrophobic barriers, electrostatic charges, low relative humidity, low or sequestered nutrient levels, endogenous microbial flora, and cross-linked proteins that contribute to a stiff cuticle (St Leger, 1991). Successful pathogenic fungi must also thwart infection-induced responses such as melanization and hemocyte activation (Pendland et al., 1993; Riley, 1997). The overall process of arthropod infection by pathogenic fungi involves many steps (Charnley and St Leger, 1991; Holder and Keyhani, 2005; Lewis et al., 2009; Wanchoo et al., 2009) that include complex systems for (1) finding (likely via passive mechanisms) the appropriate insect host(s), (2) adhering to the exoskeletal substrata, (3) evading host defenses, (4) penetrating and degrading the cuticle, (5) transporting to the cytoplasm and catabolizing necessary nutrients (carbon/nitrogen, external products of the degradation), and (6) dispersing from the catabolized host(s). Infection involves the production of specialized infection structures (appressoria), penetration of the cuticle and surrounding tissues by elongating hyphae (reaching the hemolymph), and the production of single celled hyphal bodies or blastospores within the hemolymph that are able to evade the host immune cells (Hung and Boucias, 1992; Pendland et al., 1993; Kurtti and Keyhani, 2008; Bidochka et al., 2010).

Progress has been made in uncovering some of the molecular and biochemical determinants of *B. bassiana* virulence. These include descriptions of suites of hydrolases, including proteases, lipases, and phosphatases and the production of numerous toxic metabolites such as beauvericins, oosporein, and oxalic acid. However, little is known concerning the degradation and/or penetration of the initial barrier that must be overcome for successful infection to occur, in particular the hydrocarbons that constitute the insect epicuticle or waxy layer.

## Biosynthesis of insect hydrocarbons

**Figure 2:**
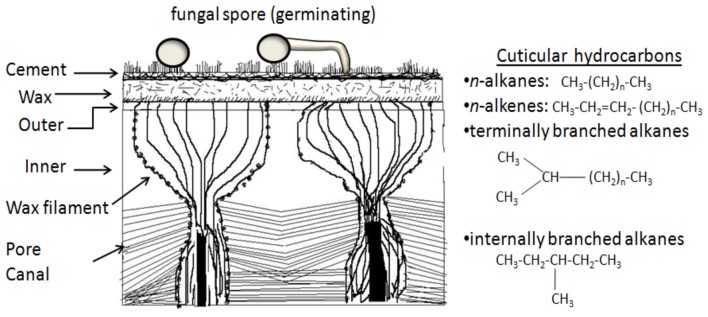
**Initial aspects of the fungal-insect interaction: the insect cuticle and its hydrocarbon contents.** Image adapted from (Noble-Nesbitt, 1991). The inner, outer, wax, cement, and bloom layers are often considered as the epicuticle, with the pore canals, that transverse the exo-, plus the meso-, and endocuticle portions (not shown in figure) comprising the procuticle.

Insect cuticular lipids are comprised of a diverse array of compounds with much variation in content and composition (Blomquist and Dillwith, 1985; Lockey, 1988; Buckner, 1993; Nelson and Blomquist, 1995) (Figure 2). This variation extends to the different life stages (adults, nymphs, larvae) and larval instars of each insect. The composition of surface lipids has profound consequences impacting ecological and behavioral aspects of the insect. Aside from acting as a barrier to desiccation and potential microbial pathogens, surface hydrocarbons contribute to numerous biochemical, physiological, and semiochemical (behavior and signaling) functions. These include roles as species, nest mate, and caste recognition cues and as a reservoir for a suite of pheromones responsible for sexual attraction, epideictic (insect display behavior), territorial markers, alarm, recruitment, chemical defense, and thermoregulation (Blomquist et al., 1987; Singer, 1998; Howard and Blomquist, 2005). Additional roles involve predator–prey and parasitoid-host interactions, mimicry and camouflage (Howard, 1993; Dettner and Liepert, 1994). Cuticular lipids include compounds with antifungal activity and components toxic to entomopathogenic fungi (Koidsumi, 1957; Smith and Grula, 1982; Saito and Aoki, 1983; Golebiowski et al., 2008). Hydrocarbons, mainly *n*-alkanes, alkenes and methyl-branched chains, are the most common epicuticular lipids. In insects, hydrocarbon are synthesized from fatty acids via an elongation-dexcarboxylation pathway which comprises (1) elongation of fatty acyl-CoAs, (2) fatty acids reduction to aldehydes by acyl-CoA reductases, and (3) conversion of fatty aldehydes to alka(e)nes with one less carbon, in an oxidative descarbonylation process catalyzed by cytochrome P450 enzymes (Blomquist et al., 1987, 1993; Nelson, 1993; Qiu et al., 2012). Cuticular hydrocarbons appear to be synthesized in oenocytes, large specialized cells rich in endoplasmic reticulum (ER) and mitochondria. Depending upon the insect species, oenocytes can be found within the epidermis, the peripheral (subcuticular) fat body or the hemocoel (Schal et al., 1998; Bagnères and Blomquist, 2010). Significant aspects of the export and deposition of hydrocarbons on the insect surface remain obscure particularly since it appears that certain parts of the insect do not synthesize hydrocarbons. It is known, however, that some hydrocarbons are transported after synthesis (presumably in the oenocytes) likely via the hemolymph to sites of deposition by reusable lipoproteins known as lipophorins, which shuttle the hydrocarbons among tissues without entering cells (Chino and Kitazawa, 1981; Van Heusden et al., 1991). Several hemolymph lipophorins have been characterized which are capable of binding newly synthesized hydrocarbons from oenocytes to the epicuticle (Gu et al., 1995; Schal et al., 1998). However, as mentioned, the mechanism of uptake, crossing of the integument, and deposition and/or assembly on the epicuticle remains unknown (Schal et al., 1998; Bagnères and Blomquist, 2010).

## Degradation of insect epicuticlular hydrocarbons by entomopathogenic fungi

The initial interaction between the fungal infectious propagule, i.e., spores, conidia, or blastospores, and the insect host occurs at the level of the insect epicuticle. It is likely that insect pathogenic microbes such as *B. bassiana* are able to recognize, assimilate and/or alter specific hydrocarbons, which in turn can impact the behavior and ecology of the insect host. Thus, an intriguing corollary to the pathogen-insect interaction is that the action of the fungus via enzymatic modification/degradation of insect cuticular compounds can have a dramatic effect on insect behavior. For instance, by degrading specific pheromones, behaviors such as grooming or nest mate recognition can be modified. Either the host or the pathogen could exploit these effects, i.e., target insects may detect infected individuals as foreign and quarantine/eliminate them (benefiting the host) or induction of behaviors such as enhanced grooming might act as a means for increased dissemination of the pathogen (although grooming typically helps eliminates microbes).

As described above, hydrocarbons, especially *n-alkanes*, *n-alkenes*, and methyl-branched chains, represent one of the major components of the epicuticle and have been extensively studied (Blomquist and Dillwith, 1985; Blomquist et al., 1987; Lockey, 1988). Insect hosts for examining fungal mediated alkane degradation include the grasshoppers, *Schistocerca americana* (Drury) and *Melanoplus sanguinipes* (Fabricius). The alkane component of the surface lipids of *S. americana* ranges from 25 to 35% of the total hydrocarbons present, covering chain lengths from C_23_–C_35_. Odd chain hydrocarbons predominate, with the major components being C_25_, C_27_, C_29_, C_31_, and C_33_ (Lockey and Oraha, 1990; Espelie et al., 1994). Similar values have been reported for the surface lipid composition of *M. sanguinipes* with C_27_ and C_29_ predominating, but also including C_23_ (Gibbs et al., 1990; Gibbs and Mousseau, 1994). *B. bassiana* can grow on most of these alkanes as a sole source of carbon.

Alternations in hydrocarbon content during fungal infection of various insects have been noted (Lecuona et al., 1991; Jarrold et al., 2007). Differences in the hydrocarbon content of the waxy layer can have profound effects on fungal pathogenesis. Some hydrocarbons inhibit spore germination, while others stimulate germination and growth (Smith and Grula, 1982; Saito and Aoki, 1983). Cuticular hydrocarbons can also promote (Boucias and Pendland, 1984; Boucias et al., 1988) or inhibit (Lord and Howard, 2004) fungal attachment to cuticle, and specific components may act as chemical inducers for the production of penetrant germ tubes on hosts (Kerwin, 1984; Latge et al., 1987). Spore germination and hyphal growth on insect lipids using pathogenic and non-pathogenic *Beauveria* strains toward the European common cockchafer (*Melolontha melolontha* L.) revealed inhibition of growth of the non-pathogenic strain by cuticular pentane extracts derived from the cockchafer, whereas no inhibition of growth of the pathogenic strains was observed (Lecuona et al., 1997). Pentane extracts of two closely related tick species, one highly susceptible to *B. bassiana* (*Amblyomma maculatum* Koch) and the other somewhat resistant to fungal infection (*A. americanum* L.), revealed inhibition of fungal germination in the case of *A. americanum* but good growth on the *A. maculatum* extracts (Kirkland et al., 2004a). *B. bassiana* has been shown to utilize several insect hydrocarbons including aliphatic and methyl branched alkanes (Napolitano and Juarez, 1997). C_28_ and C_24_ alkanes were degraded by *B. bassiana* mainly into free fatty acids, phospholipids, and acylglycerols, with alkane grown cells producing *n*-decane as a volatile organic compound as a by-product of the β-oxidation reactions (Crespo et al., 2000, 2008). Similarly, the major components of the larvae of the blood-sucking bug *Triatoma infestans* Klug (an important vector of human disease causing microbes) epicuticle includes C_29_, C_31_, and C_33_ are able to promote *B. bassiana* growth (Napolitano and Juarez, 1997). Radiolabeled hydrocarbons have been used to investigate the catabolic pathways of alkane degradation in *B. bassiana*, and these data support a degradative pathway involving β-oxidation by a cytochrome P450 enzyme system, followed by peroxisome mediated successive transformations to yield the appropriate fatty acyl CoA as further described below (Pedrini et al., 2006, 2007). Alkane growth has been linked to increased virulence, with *B. bassiana* cells grown on alkane containing media displaying a dramatic 2–4-fold increase in mortality against the bean weevil *Acanthoscelides obtectus*, when compared to cells grown on glucose (Crespo et al., 2002). These data indicate that *B. bassiana* mediated alkane degradation represents a key metabolic pathway that is linked to the entomopathogenic nature of the fungus.

## *n*-alkane assimilation in fungi

Little is known concerning how alkanes are taken up and transferred into cells by fungi. Active transport appears to be involved and *n*-alkane uptake experiments performed in *Cladosporium resinae* in the presence of metabolic uncouplers indicate that the uptake of alkanes in fungi comprises (1) passive adsorption to the outer cell surface where long hair-like structures are formed upon alkane binding, also seen in alkane-grown *Candida tropicalis* and *B. bassiana* cells (Kappeli et al., 1984; Juárez et al., 2004) and (2) an energy-requiring transfer of the unmodified alkane into the cytosol (Lindley and Heydeman, 1986). Typically, after binding the cell surface, *n*-alkanes are solubilized in order of increasing molecular weight (Goma et al., 1973; Reddy et al., 1982; Cameotra et al., 1983; Lindley and Heydeman, 1986). Subsequently, these alkanes are shuttled into the cell inside of membrane-bound vesicles likely by a process of pycnocytosis (Meisel et al., 1973; Cooney et al., 1980; Lindley and Heydeman, 1986). Although unclear, the role of these membrane-bound vesicles is thought to provide continuous input of alkanes while avoiding the potential toxicity of insoluble alkanes floating in the cytosol (Lindley and Heydeman, 1986).

**Figure 3:**
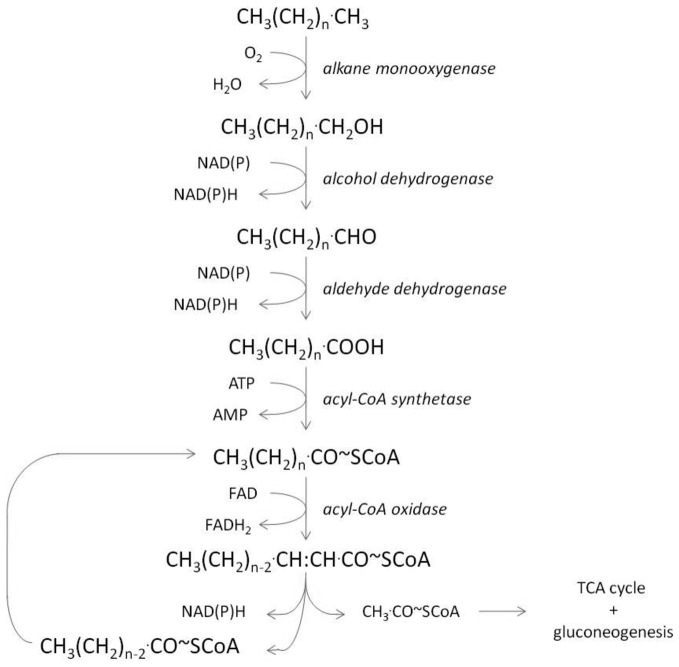
**Proposed main catabolic pathway for alkane degradation**.

**Figure 4:**
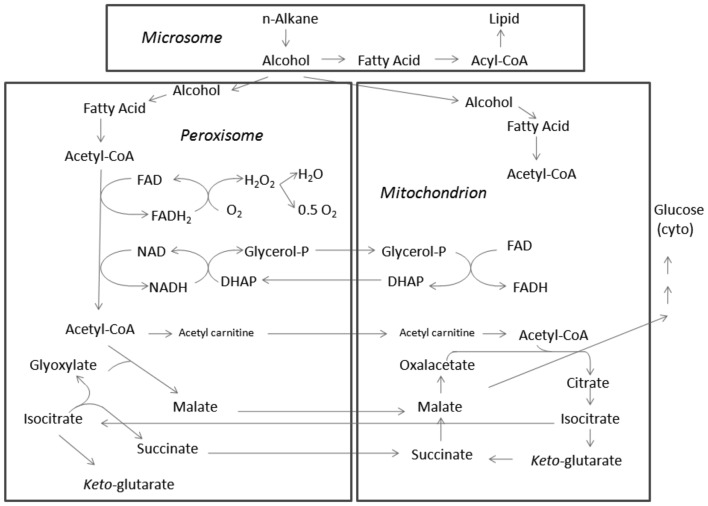
**Sub-cellular distribution of hydrocarbon catabolic enzymes**.

Many fungi have developed metabolic systems to assimilate *n*-alkanes as carbon sources via the activities of cytochrome P450 mono-oxygenases (Figure 3) (Lindley, 1995; Van Beilen et al., 2003; Singh, 2006; Rojo, 2010). However, while much is known about these enzymes in *n*-alkane assimilating yeasts, such as *Candida maltosa* and *Yarrowia lipolytica*, their orthologs in filamentous fungi have not yet received adequate attention. In yeasts cytochrome P450ALKs (alkanes), belonging to the CYP52 family (Nelson, 2009), are thought to catabolize *n*-alkanes. Where examined, in yeasts, the pathway starts with terminal hydroxylation of alkanes to fatty alcohols by P450ALKs in the ER, and further oxidation to fatty aldehydes either by the fatty alcohol dehydrogenase (FADH) in the ER or by fatty alcohol oxidase (FAOD) in the peroxisome. Whether in the ER or peroxisome, fatty aldehydes are oxidized by fatty aldehyde dehydrogenases (FALDHs) to fatty acids that are further activated by acyl-CoA synthetases (ACS I and/or ACS II). The activated fatty acids are then utilized in membranes or storage lipids, or degraded in the peroxisome via β-oxidation to yield acetyl-CoA (Fickers et al., 2005). Similarly, in filamentous fungi, the hydroxylation of the terminal methyl group of *n*-alkanes is carried out in the ER by (specific) cytochromes P450s that are coupled to general NADPH-cytochrome P450 reductases. The resultant fatty alcohol can also be catabolized to activate fatty acid in the mitochondrion in addition to ER and peroxisome. The activated fatty acids are catabolized to acetyl-CoA by β-oxidation in the peroxisome and/or the mitochondrion (Figure 4).

In both yeast and filamentous fungi, although there has been little examination of the central pathways of alkane metabolism, it is considered to involve a predominantly amphibolic tricarboxylic acid cycle with high glyoxylate bypass activity and gluconeogenesis. Such a metabolic pathway results in highly reduced co-enzyme and acetyl-CoA production, with the latter compound feeding anabolic pathways. Alternate pathways include diterminal or subterminal oxidation of alkanes whose products are ultimately assimilated via β-oxidation reactions, where in the latter case a mixture of secondary alcohols can be formed which are first metabolized to yield the corresponding primary alcohols (which then undergo dehydrogenation) and organic acids (Figure 4). There remain, however, many unanswered biochemical questions regarding *n*-alkane assimilation by fungi.

## Alkane catabolic pathway and cytochrome P450s in *B. bassiana*

*B. bassiana* is likely to contain novel enzymes due to the diverse nature and large chain lengths of the hydrocarbon constituents of the insect waxy layer. In the pre-genomic era we identified several genes implicated in alkane degradation in *B. bassiana*, by exploring our EST dataset (Cho et al., 2006a,b). These included eight cytochrome P450 genes (CYP) encoding enzymes with putative specificity for alkanes (Pedrini et al., 2010), four catalases, and long chain alcohol and aldehyde dehydrogenases (Table 1). Overall, we have identified at least 12 additional P450 genes (for a total of at least 20 P450 genes in *B. bassiana*) in EST dataset with substrates specificities for a range of compounds besides alkanes. The recent release of *B bassiana* complete genome (Xiao et al., 2012) has permitted the extension of this list of genes as follows; 77 CYP genes with families and subfamilies assigned, five catalases and at least 22 alcohol dehydrogenases and 11 aldehyde dehydrogenases.

**Table 1:** **Candidate genes involved in alkane degradation identified in the *B. bassiana* EST dataset**.

**Gene (accession No.)**	**Putative function**	**Gene knockout available?**	**Phenotype**	**References**
CYP52(X1) (GU566074)	Lipid oxidation	Yes	Decreased virulence in insect topical assays/no effect in intrahemoceol injection assays	Zhang et al., 2012
CYP655(C1) (AM409327)	Lipid oxidation	Yes	No phenotype detected thus far	This study
CYP5337(A1) (GU566075)	Lipid oxidation	Yes	No phenotype detected thus far	This study
CYP52(G11) (GU566076)	Lipid oxidation	Yes	No phenotype detected thus far	This study
CYP539(B5) (GU566077)	Lipid oxidation	No	–	–
CYP617(N1) (GU566078)	Lipid oxidation	Yes	No phenotype detected thus far	This study
CYP53(A26) (GU566079)	Lipid oxidation	Yes	No phenotype detected thus far	This study
CYP584(Q1) (GU566080)	Lipid oxidation	No	–	–
*catA* (spore-specific)		Yes	Decreased virulence, thermotolerance and UV resistance. Not tested for alkane degradation	Wang et al., 2012
*catB* (secreted)	H_2_O_2_ scavenging	Yes	Not tested for alkane degradation	Wang et al., 2012
*catP* (peroxisomal)	β-oxidation pathway	Yes	Decreased virulence. Not tested for alkane degradation	Wang et al., 2012
*catC* (cytoplasmic)	H_2_O_2_ scavenging	Yes	Not tested for alkane degradation	Wang et al., 2012
*catD* (secreted peroxidase/catalase)	H_2_O_2_ scavenging	Yes	Decreased virulence and UV resistance. Not tested for alkane degradation	Wang et al., 2012
Acyl CoA oxidase	β-oxidation pathway	No	N/A	N/A
3-oxoacyl carrier protein reductase	Biosurfactants synthesis/transport	No	N/A	N/A
ADH-2 FALDH	Long-chain alcohol and aldehyde dehydrogenase	No	N/A	N/A

From genome analyses, fungi have a large diversity in P450 content. Yeasts such as *S. cerevisiae*, *C. albicans*, and *Y. lipolytica* contain 3, 10, and 17 identified P450 ORFs, respectively. Filamentous fungi such as *Neurospora crassa*, *Magnaporthe grisea*, and *Fusarium oxysporum*, contain 43, 139, and 170 putative P450 enzymes, respectively, and the basidiomycetes, *Phanerochaete chrysosporium* and the brown rot fungus, *Postia placenta* contain 145 and 353, P450 ORFs, respectively (Source: Fungal Cytochrome P450 DataBase). Other insect pathogens, such *as Metarhizium robertsii* and *M. acridum*, have 123 and 100 CYP genes, respectively (Gao et al., 2011). Thus from genomic analyses, fungi and plants appear to contain the largest complements of CYP genes, probably due to the diversity of both primary and secondary metabolism, and xenobiotic transformation and detoxification pathways.

Within the P450 superfamily, genes are assigned into families and subfamilies based mainly on amino acid sequence identity. Genes are assigned to families when they share greater than 40% amino acid identity with reference sequences and are assigned to subfamilies when they are more than 55% identical (Nelson et al., 1996). A higher order for grouping P450 genes, called the clan, has been proposed and applied to studies of P450s from different Kingdoms. The introduction of clan categories attempts to group genes based on robust phylogenetic relationships. Genes within a clan likely diverged from a common ancestor gene (Nelson, 1999) and may share common functions (Nelson, 1998). In fungi, few phylogenetic studies using P450s have been reported. In the basidiomycete *P. chrysosporium*, 12 CYP families were classified into 11 clans (Doddapaneni et al., 2005), whereas the sum of the four ascomycetes *Aspergillus nidulans*, *N. crassa*, *F. graminearum*, and *M. grisea* has a total of 376 P450 genes that were assigned to 168 families clustered into 115 clans (in average 42 families and 29 clans per species) (Deng et al., 2007). The availability of the *B. bassiana* genome has contributed to the further annotation of the diversity of fungal P450s; from our analysis at least 15 sequences represent the founding members of a new cytochrome P450 family (20% of total P450s), and 21 genes (27%) appear to represent the first members of new subfamilies (Table 2).

**Table 2:** **Cytochrome P450 monooxygenase genes (CYP) in *B. bassiana***.

**CYP clan**	**CYP family**	**CYP subfamily**
CYP 54	CYP 503	B1
CYP 504	CYP 504	A6, B10, E1, E5
CYP 505	CYP 505	A1 (CYPOR), A2, D4
nd	CYP 5060	A1
CYP 531	CYP 5080	B3
CYP 56	CYP 5099	A1
CYP 51	CYP 51	F1, F2
CYP 52	CYP 52	X1, T1, G6, G8, G11
nd	CYP 5202	A1
CYP 526	CYP 526	H1
nd	CYP 5262	A3
CYP528 (kr)/53 (dn)	CYP 528	A4
nd	CYP 5280	A1P
nd	CYP 5282	A1
nd	CYP 5293	A1, A2
CYP 53	CYP 53	A11, A26
nd	CYP 5337	A1
CYP 534	CYP 534	C2
CYP 537 (kr)/53 (dn)	CYP 537	A4
CYP 52	CYP 539	B1, B5
CYP 540	CYP 540	B16
CYP 505	CYP 541	A2
CYP 58 (kr)/53 (dn)	CYP 542	B1, B2, B3
CYP 548 (kr)/53 (dn)	CYP 548	A5
CYP 58	CYP 551	C1
CYP 56	CYP 56	C1
CYP 65 (kr)/53 (dn)	CYP 561	D2P
CYP 507 (kr)/53 (dn)	CYP 570	A1, H1, E2,
CYP 578	CYP 578	A2
CYP 58	CYP 58	A3
CYP 52	CYP 584	D4, E2, E7, G1, Q1
CYP 59	CYP 586	B1
nd	CYP 6001	C8
nd	CYP 6003	A1
nd	CYP 6004	A2
CYP 61	CYP 61	A1
CYP 547	CYP 617	A1, A2, N1
CYP 533	CYP 620	C2, D1
CYP 533	CYP 621	A2
CYP 559	CYP 623	C1
CYP 578 (kr)/53 (dn)	CYP 625	A1
nd (kr)/53 (dn)	CYP 628	A2
CYP 639	CYP 639	A3
CYP 645	CYP 645	A1
CYP 65	CYP 65	A1, T7
CYP 52	CYP 655	C1
CYP 550	CYP 660	A2
CYP 68	CYP 68	N1
CYP 58 (kr)/53 (dn)	CYP 682	H1, N1
CYP 58 (kr)/53 (dn)	CYP 684	A2, B2
	Total	77
	New CYP family	15 (19.5%)
	New CYP subfamily	21 (27.3%)

The genes with experimental evidence of involvement in both hydrocarbon and insect lipid degradation are underlined.

Source: Xiao et al. (2012). Kr, It was generated via the implemented pipeline in the Fungal Cytochrome P450 Database; dn, Nelson's curation; nd, not determined (neither by kr nor dn).

**Figure 5:**
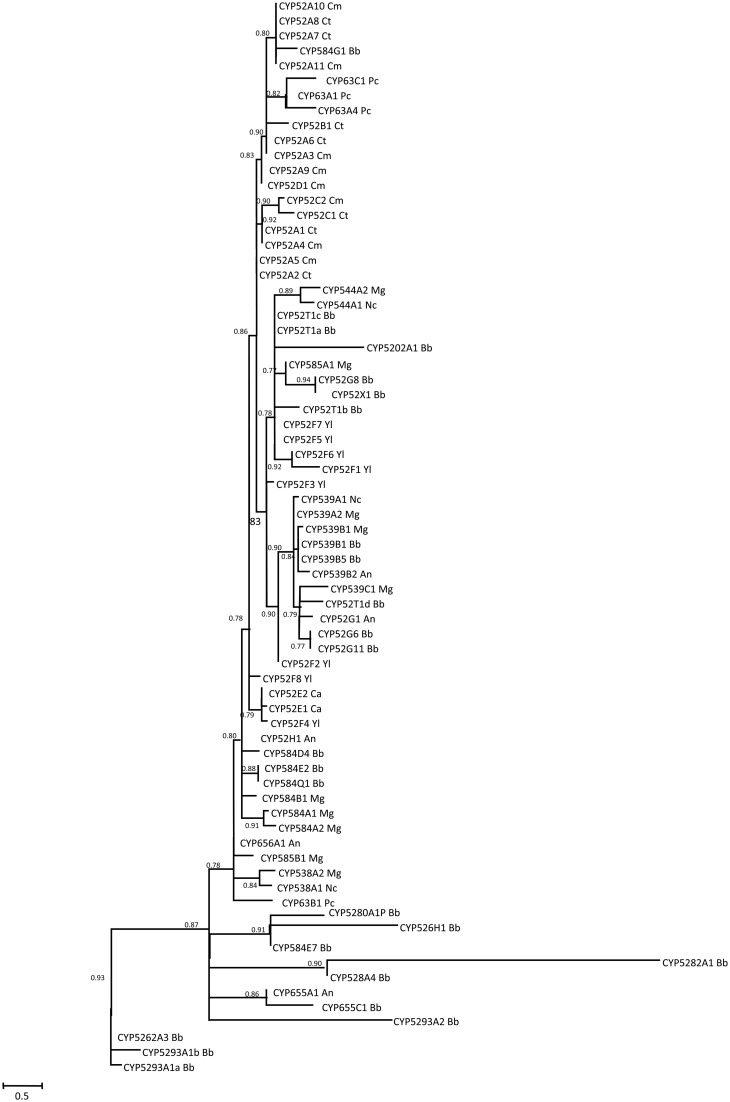
**Maximum likelihood phylogenetic tree of clan 52 cytochrome P450s.** The putative conserved domains for each protein were picked up from the conserved domain database (CDD) (Marchler-Bauer et al., 2011), and aligned using MUSCLE. The multiple sequence alignment was cured with Gblocks. PhylML was used to build the tree. All the analyses were performed at the online platform Phylogeny.fr (Dereeper et al., 2008, 2010). Numbers at nodes indicate SH-like branch support. Scale bar indicates number of amino acid substitutions per site. Amino acid substitution model John Taylor Thornton (JTT).

**Figure 6:**
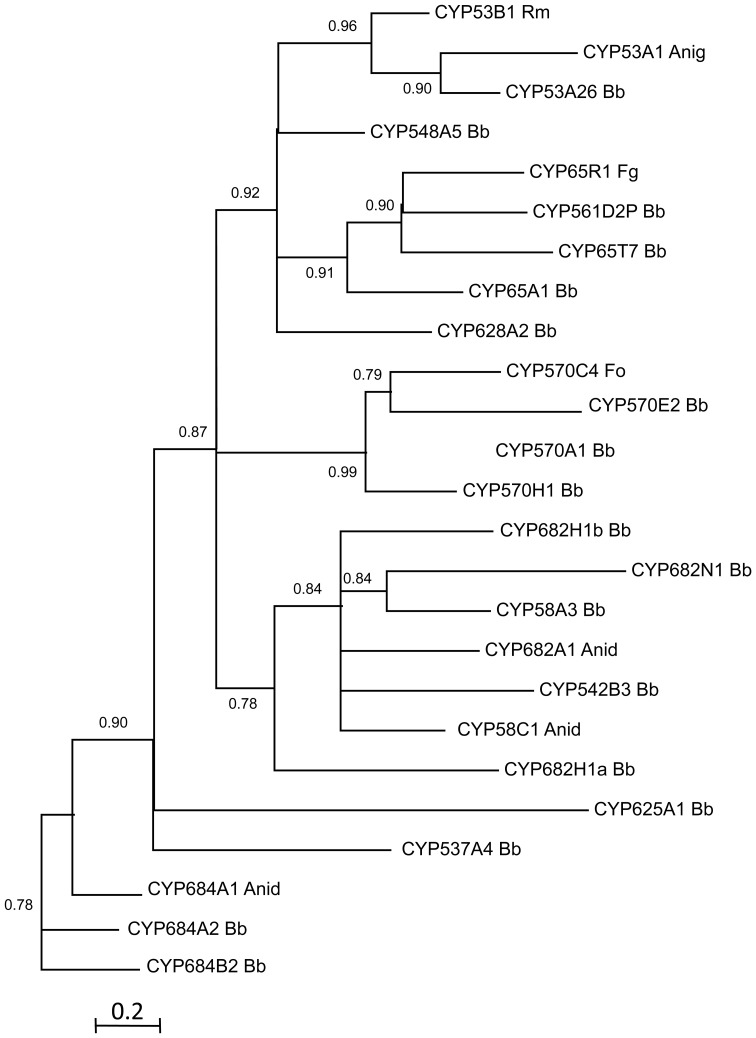
**Maximum likelihood phylogenetic tree of clan 53 cytochrome P450s**.

Genome mining of *B. bassiana* indicates the presence of two clans that represent 45.5% of total P450 genes: CYP52 and CYP53 clans (Table 2). The CYP52 family, part of the CYP52 clan, was originally identified in alkane assimilating yeast, and identified to have a role in terminal hydroxylation of *n*-alkanes and fatty acids. This clan includes the highest gene number per family in *B. bassiana*, with five genes in each of the families CYP52 and CYP584 (the CYP584 family is part of the CYP52 clan). Two genes, belonging to the CYP52 family, have been (partially) characterized in *B. bassiana*, with evidence that they participate in both hydrocarbon and insect lipid degradation (Pedrini et al., 2010; Zhang et al., 2012). However, BbCYP584Q1 showed little to no induction in any of the alkane growth conditions examined (Pedrini et al., 2010). Other members of this clan (BbCYP539B5 and BbCYP655C1) are induced in the presence of C_16_, C_20_, C_24_, C_28_, and *T. infestans* lipid extract carbon sources (Pedrini et al., 2010). A phylogenetic analysis of the CYP52 clan revealed that these genes fall into discrete clusters (Figure 5). The significant divergence in amino acid sequence observed may indicate substrates beyond alkanes and/or likely reflects distinct biological roles for subsets of these proteins. The CYP53 family was first described as including benzoate 4-hydroxylases in *A. niger* and *Rhodotorula minuta*. *B. bassiana* has several candidate genes that fall within the CYP53 clan (Figure 6). Amongst these, BbCYP53A26, is induced in the presence of various hydrocarbons and insect lipids (Pedrini et al., 2010), suggesting that this fungus employs a differentiated strategy for hydrocarbon-assimilation using a variety of enzyme classes. However, not all identified cytochromes may be directly involved in lipid assimilation. For example, *B. bassiana* CYP655C1 (CYP52 clan) expression was strongly induced by hydrocarbons and insect lipids, but it appears to be involved in tenellin synthesis (with aromatic intermediates) (Doddapaneni et al., 2005), indicating that lipid may act as signals for the biosynthesis of select fungal secondary metabolites. In the basidiomycete, *P. chrysosporium*, seven members belonging to the CYP63 family have been identified which all can be classified under the CYP52 clan. All seven genes showed transcriptional induction with alkanes, mono-aromatic and polycyclic aromatic hydrocarbons, and also alkyl-substituted aromatics compounds (Yadav et al., 2006).

## Cytochrome P450 expression in *B. bassiana*

The expression pattern of eight *B. bassiana* cytochrome P450 enzymes has been examined under a variety of conditions (Pedrini et al., 2010). Cells grown in minimal media containing either C_16_, C_24_, or C_28_ as the sole source of carbon showed significant induction of several of the cytochrome P450 genes by growth on specific alkanes as compared to glucose grown cells. Of the set of *B. bassiana* cytochrome p450 examined, enzymes belonging to the families CYP52(X1) and CYP617(N1) showed only slight to moderate induction in the alkanes tested. In contrast, Bb-CYP655(C1) and Bb-CYP52(G11) were induced >200-fold in all alkanes tested (C_16_, C_24_, and C_28_), with Bb-CYP655(C1) displaying maximal induction by C_24_. Bb-CYP5337(A1) showed slight induction when grown on C_16_ and C_24_, but >200-fold when grown on C_28_, indicating that it may be important for oxidation of longer chain length alkanes. Bb-CYP53(A26) displayed only minor induction when grown on C_16_, but >200-fold by C_24_ and C_28_. Bb-CYP584(Q1) displayed only minor to moderate induction when grown on C_16_ and C_28_, but >200-fold by C_24_. These data support a model indicating the presence and importance of a suite of P450 enzymes with overlapping but distinct substrate and expression specificities, particularly since P450s are well-known to be induced by their substrates (Montellano, 2005).

Thus far, the expression patterns of these P450s have only been examined after fungal growth on insect-derived lipids from the blood-sucking bug, *T. infestans* (Pedrini et al., 2010). Similar to what was observed for the pure alkanes, three distinct induction profiles were noted: Bb-CYP655(C1) and CYP617(N1) were highly induced (>200-fold), Bb-CYP52(X1), CYP5337(A1), and CYP53(A26) were moderately induced (>30-fold), and Bb-CYP539(B5), CYP52(G1), and CYP584(Q1) displayed low to no induction, i.e., 12-fold, 4-fold, and no induction, respectively when grown in *T. infestans* cuticular lipid extracts. Since the content and structure of hydrocarbons and lipids shows considerable variation not only between diverse insect but sometimes between the various life stages of a particular insect, it is intriguing to hypothesize that hydrocarbon assimilating cytochrome P450s may act as partial specificity factors, helping to account for the broad host range of entomopathogenic fungi such as *B. bassiana*. Thus, such an idea would predict that individual members of *B. bassiana* P450 (lipid assimilating) repertoire would differentially contribute to the pathogenic process depending upon insect target. Further examination of the expression profiles of the P450s after growth on different insects would help shed light on this issue. In addition, knowledge concerning the contributions and/or importance of individual P450s to the ability of the fungus in targeting specific insects could be used to manipulate, i.e., increase, the virulence of the fungus toward those targets, e.g., by increasing the expression levels of critical P450s.

## Genetic dissection of the alkane pathway: cytochrome P450s

To date, the role of a single cytochrome P450, Bb-CYP52(X1) has been investigated genetically in *B. bassiana* (Zhang et al., 2012). A targeted gene knockout mutant of Bb-CYP52(X1) did not display any noticeable growth defects on any substrates tested including alkanes ranging from C_9_–C_28_, fatty acids, e.g., oleic, linoleic, stearic, palmitic, myristic, and lauric acids, or in media containing olive oil. Intriguingly, neither wild type nor the mutant strain was able to grow on pelargonic acid. A grasshopper wing assay in which fungal spores are deposited onto dissected wings and germination/fungal growth was measured, however, revealed a difference between the wild type and ΔBb-CYP52(X1) strains. Germination of the mutant strain on the grasshopper wings was 50% lower than the wild type or complemented mutant strains, the latter representing a mutant strain in which the wild type gene was retransformed into the fungus under control of its endogenous promoter. Perhaps the most interesting results dealt with the virulence of the mutant strain in insect bioassays. As previously mentioned, *B. bassiana* infects target host via (random) attachment to the cuticle, followed by germination, hyphal growth along the surface, and penetration through the cuticle into the insect hemocoel. Topical application of fungal conidia (spores) represents that “natural” route of infection, and experiments performed using the Greater Waxmoth, *Galleria mellonella*, indicated a 25–50% reduction in virulence (time to kill, LT_50_ value) in the ΔBb-CYP52(X1) strain as compared to the wild type and complemented strains. Cuticle penetration can be bypassed, however, by directly injecting the fungal cells into the insect hemoceol. In such experiments, i.e., intrahemoceol injection assays, the mutant strain was as virulent as the wild type parent. These data support a hypothesis that certain cytochrome P450 enzymes are important for cuticle penetration events, presumably via assimilation or detoxification of cuticular substrate for the enzyme, but that these P450s are not required for post-penetration events once the cuticle has been breached. An important piece of the puzzle, however, remains obscure, namely, since P450s are ER-derived membrane bound proteins, how are their (cuticular) substrates accessed and/or transported to the proteins?

We have also constructed targeted gene knockouts of the six out of eight of the other identified cytochrome P450 enzymes implicated in insect hydrocarbon degradation (Table 1). However, to date, no phenotype with respect to growth or germination on lipids or virulence has been noted for any of these mutants (data not shown). These results may not be too surprising for several reasons. First, due to the potential redundancy and/or (partial) overlapping substrates specificities of these enzymes single gene knockout like we have constructed may not display any noticeable phenotypes. This has been also observed in *Y. lipolytica*, where the abundance of paralog genes encoding for alkane degradation proteins, makes it difficult to unravel the function and the physiological substrate(s) of individual alkane degradation proteins (Takai et al., 2012). Second, the virulence of these strains have only been examined with respect to a single insect target (*G. mellonella*), it is possible that some of these enzymes may have substrates found on other targets not present on *G. mellonella*. Thus, assaying a diversity of target insects may reveal differential contributions of various P450s to the pathogenic process toward specific insects. If properly demonstrated, this would support the idea that P450s can act as insect target specificity factors, contributing to the broad host range nature of *B. bassiana*.

## Biochemical characterization of *B. bassiana* cytochrome P450s

*B. bassiana* CYP52(X1) has been expressed in a yeast (*Saccharomyces cerevisiae*) heterologous expression system and its activity examined in yeast-derived microsomal extracts (Zhang et al., 2012). This yeast system has been optimized for expression of cytochrome P450s and contains elevated amounts of the needed companion reductase for activity (Pompon et al., 1996). Intriguingly, although a low spin heme spectrum was detected in yeast microsomes isolated from expression-induced cells harboring the Bbcyp52x1 plasmid construct, no CO difference spectral shift, a tell-tale biophysical signal for cytochrome P450 content, was observed. The (ER-derived) microsomes, however, displayed NADPH-dependent oxidation of a number of substrates under conditions in which no activity was detected in control microsomes derived from wild type yeast cells or cells transformed with an empty vector. Use of radiolabeled lauric and oleic acids followed by GC/MS analysis confirmed CYP52X1 mediated NADPH-dependent regioselective addition of a terminal hydroxyl to both substrates. TLC analysis of reaction products using a variety of other fatty acid substrates revealed that CYP52X1 displayed highest activity against C12:0, C14:0, and epoxy stearic acid, 4–8-fold lower activity against C16:0, C18:1, and C18:2, and little to no activity toward C9:0 and C18:0 (Zhang et al., 2012).

**Figure 7:**
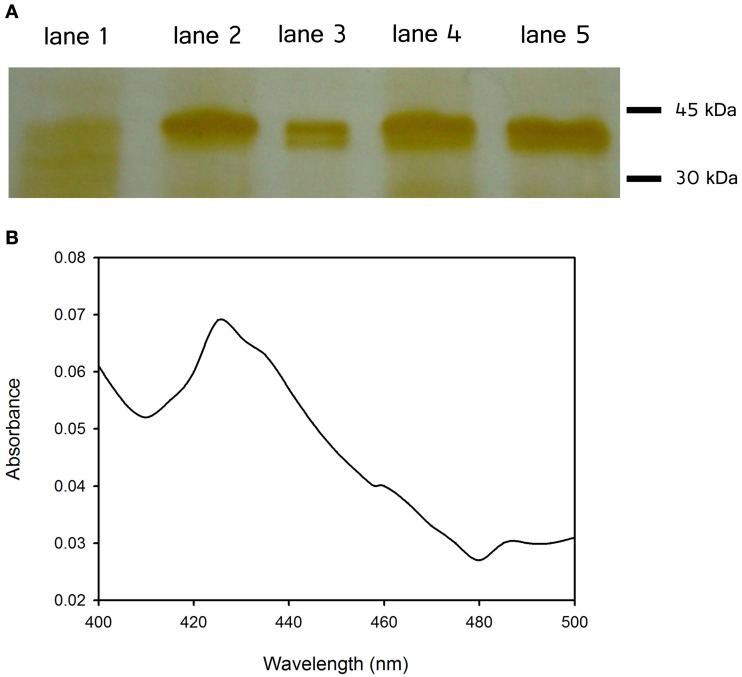
**(A)** SDS–PAGE of partial purified proteins using immobilized metal ion chromatography (Ni^2+^ IMAC): vector without insert (lane 1), CYP52X1 (lane 2), CYP5337A1 (lane 3), CYP617N1 (lane 4), and CYP53A26 (lane 5). Examined proteins were expressed in the yeast *Saccharomyces cerevisiae* WAT11. The cells were transformed with the lithium acetate method (Ito et al., 1983) for microsome isolation purposes. Proteins were solubilized with 10% w/v sodium cholate and loaded onto the column. **(B)** CO difference spectrum of CYP53A26: Sodium dithionite and CO were added to the purified protein, and absorbance between 400 and 500 nm was measured.

Additional *B. bassiana* proteins, namely, CYP5337A1, CYP617N1, CYP53A26, and CYP584Q1 have also been expressed in the same yeast system. These constructs contained N-terminal his-tags which have been used for partial purification using immobilized metal ion chromatography (Ni^2+^ IMAC) (Figure 7). Of the proteins examined thus far, only CYP53A26 showed a CO spectrum corresponding to the oxidized protein, including a peak at 426 nm and a small shoulder at 450 nm (Figure 7). The rest of the proteins showed no CO difference spectral shift when dithionite was added (data not shown). Poor or atypical CO spectra have been reported for a number of cytochrome P450s including some plant P450s that have weak affinity for CO (Lau et al., 1993), and P450 19A1 (human aromatase) which does not bind CO (Harada, 1998; Gartner et al., 2001). Our data suggest that the number of P450s with atypical CO spectra is greater than what is currently thought.

## Fatty alcohol and aldehyde dehydrogenases

Upon terminal hydroxylation of alkanes to fatty alcohols by P450ALKs, fatty alcohol and aldehyde dehydrogenases (FADHs and FALDHs) oxidize the fatty alcohols to fatty aldehydes and fatty acid, respectively. There have been few reports examining fungal very long chain alcohol and aldehyde dehydrogenases, and little is known concerning their potential role in the oxidation of the fatty alcohol products of P450 activity. Dehydrogenases, however, are considered to yield the fatty acids that feed into the β-oxidation pathway. Fatty alcohol dehydrogenases (FADHs) are mainly linked to the biosynthesis of mannitol, an important intracellular carbohydrate factor that participates in stress tolerance and has even been linked to virulence in certain fungal pathogens. *M. anisopliae* has 17 zinc-containing alcohol dehydrogenases (Gao et al., 2011). FALDHs, responsible for the oxidation of various aldehydes to their corresponding carboxylic acids are widely distributed in filamentous fungi. Although little empirical data is currently available, it is postulated that in entomopathogenic fungi specific FALDHs may exist with substrate specificity for the aldehydes present or generated during cuticular hydrocarbon assimilation. Two *M. anisopliae* FALDHs have thus far been examined in relation with cuticle degradation, and these have been shown to be up-regulated in when the fungus is grown in the presence of insect cuticles as compared to growth in sugar-rich media (Freimoser et al., 2005). It is likely therefore that the observed induction of these genes in the presence of insect cuticle is a consequence of the availability of aldehydes resulting from the oxidation of cuticular hydrocarbons.

In the *Aspergillus nidulans* ethanol utilization pathway, FALDH and FADH are co-regulated, at the transcription level, via the *alc* regulon (Flipphi et al., 2001). Aside from ethanol and acetaldehyde, other inducers of the *alc* system including amino acids and other aliphatic alcohols have been identified. However, it is thought that the real physiological inducers are aldehydes, and that the other examined compounds are first converted to their corresponding aldehydes in order to act as inducers (Flipphi et al., 2001). Analysis of the promoter region of the FALDH *ald1* of the mycorrhizal fungus *Tricholoma vaccinum* revealed the presence of five putative stress response elements (STREs) (Asiimwe et al., 2012). These elements have also been detected in the promoters of several *B. bassiana* CYP genes (Pedrini et al., 2010), suggesting common induction/regulatory compounds and pathways function to co-regulate FALDH, FADH, and CYP activities. It should be noted that alternative pathways also exist; the alkane-assimilating yeast *C. maltosa* is able to catalyze the production of a cascade of mono-oxidation products followed by di-terminal oxidation of substrates to yield α −ω acids. These reactions can be catalyzed by a single P450 enzyme and include both alcohol and aldehyde intermediates but do not use the corresponding dehydrogenases (Scheller et al., 1998). Although no evidence exists for such pathways in *B. bassiana*, at this time they cannot be excluded.

Feeding of hydrocarbon assimilation products into central metabolic pathways (Figure 4) may also contribute to the *B. bassiana* pathogenic lifecycle. Isocitrate lyase (ICL) and malate synthase (MLS) are not only up-regulated during growth on two-carbon compounds including acetate and ethanol, but also during growth in insect hemoplymph (Padilla-Guerrero et al., 2011). In *M. anisopliae*, *icl* is up-regulated during the initial infection stage of appressoria formation as well as during late host growth events when the fungi are engulfed by insect heamocytes, highlighting the contribution of the glyoxylate cycle in pathogenesis (Padilla-Guerrero et al., 2011). Production of metabolic acids by entomopathogenic fungi, potentially resulting from cuticular hydrocarbon recognition and assimilation cues, can also directly participate in insect virulence; and citrate, formate, and oxalate have been shown to contribute to *B. ba*ssiana virulence (Bidochka and Khachatourians, 1991; Kirkland et al., 2005). Oxalate production in particular contributes to pathogenesis via acidification of host tissues, sequestration of metal ion such as calcium, magnesium, manganese, and iron and could inhibit or disrupt host defense responses (Kirkland et al., 2005).

## Catalases

Fatty acids are completely catabolized through β-oxidation. Peroxisomal proliferation together with a marked induction of the β-oxidation system has been related to alkane-growth adaptation in *B. bassiana* (Crespo et al., 2000). The typical peroxisomal marker enzyme is catalase, a hemoprotein that decomposes excess H_2_O_2_ produced by β-oxidation. Peroxisomal catalase induction was observed in alkane-grown *B. bassiana*, a high increment in the catalase activity (14-fold) was measured in these fungi compared with control cultures grown in complete medium (Pedrini et al., 2006). Catalase activity was also detected in the cytosolic fraction of *B. bassiana*, although this isoform was not induced in the same culture condition (Pedrini et al., 2006). Catalase induction was proposed as a simple biochemical marker to follow the course of fungal growth adaptation in insect-like hydrocarbons and was correlated with enhance virulence parameters (i.e., lower LC_50_ and/or LT_50_) (Crespo et al., 2002; Pedrini et al., 2007, 2009).

The catalase family of *B. bassiana* consists of at least five genes designed as; catA (spore-specific), catB (secreted), catP (peroxisomal), catC (cytoplasmic), and catD (secreted peroxidase/catalase). The functions of these genes and their protein products have been studied via generation of single targeted gene disruption mutant strains (Wang et al., 2012). CatB appeared to account for the predominant catalase activity produced by the fungus with ΔcatB mutants displaying ~90% decrease in total catalase activity, however ΔcatP mutants were reported to results in ~55% decrease in total catalase activity, suggesting negative pleiotropic effects between certain catalases. Intriguingly, ΔcatB (and ΔcatC) mutants displayed only minor phenotypic effects, which the authors explain by the observation of up-regulation of the other catalases in these mutants and hence potential functional redundancy. If correct, it is unclear, however, how this would explain the low (~10% of wild-type) activity seen in the ΔcatB mutant. ΔcatA strains were more theromosensitive than the wild type parent, showed a ~50% decrease in UV-B resistance, decreased virulence in insect bioassays, and conidial sensitivity to H_2_O_2_ although colony growth in the presence of H_2_O_2_ was essentially unaffected. ΔcatD strains were unaffected in thermal stability and oxidative stress in general, but did display reduced UV-B tolerance and virulence in insect bioassays. Deletion of the peroxisomal catalase (catP), the enzyme most likely to be involved in hydrocarbon assimilation pathways, resulted in increased sensitivity to oxidative stress both on the conidial germination level and during colony (vegetative) growth. Δ catP mutants were essentially unaffected in thermal sensitivity and UV-B tolerance, but displayed ~50% decrease in virulence (LT_50_) indicating that it is an important enzyme involved in the pathogenic process (Wang et al., 2012). Although alkane and hydrocarbon assimilation in the catP (or other catalase) mutants has not yet been examined, these data suggest that peroxisomal catalases might be crucial factors for adaptation to oxidative stress generated during fungal growth on insect alkanes and other hydrocarbons.

## Insect cuticular hydrocarbons synthesis and fungal hydrocarbon assimilation—an example of “red queen” co-evolution?

The co-evolutionary arms race between a pathogen and its target host has been referred to as the “Red-Queen Hypothesis,” and is taken from Lewis Carroll's Through the Looking Glass, where in the Red Queen's race “it takes all the running you can do, to keep in the same place” (Van Valen, 1973). The hypothesis is that target hosts evolve mechanisms for resistance against pathogens, and that pathogens, in turn, evolve mechanisms for surmounting the evolving host defenses. Could insect cuticular hydrocarbon synthesis be under a Red Queen selection mechanism? It is undoubtable that the insect epicuticle layer, with its complexity of hydrocarbons serves as a means for protection against abiotic stress, e.g., desiccation, heat, and even potentially UV irradiation, and that this layer serves as a platform for semiochemicals involved in insect communication and signaling. It is also clear that many of the hydrocarbons present in the epicuticle have antimicrobial properties and suppress the growth of microbes. Antimicrobial mechanisms may be passive in that these hydrocarbons are poor substrates for most microbes and that they can effectively sequester nutrients from scavenging and/or attacking parasites. However, there is ample evidence for the existence of chemically diverse species-specific antimicrobial compounds targeted for secretion to the insect epicuticle. Both such molecules must be overcome for successful pathogenesis to occur. Specific antimicrobial compounds would require detoxification and/or remediation that occur via enzymatic inactivation and/or efflux or sequestration of such molecules via multidrug-efflux systems. In this respects *B. bassiana* displays a high level of resistance to many antifungal compounds and genomic analyses has revealed a large set of detoxifying enzymes as well as efflux transporters. Insect epicuticles, however, often contain significant amounts of long chain hydrocarbons, whose purpose regarding abiotic stress and/or semiochemicals potential are obscure. However, long(er) chain hydrocarbons become increasingly difficult for microbes to assimilate and can directly inhibit the growth of many bacteria and fungi. As elongation reactions in insect hydrocarbon biosynthesis typically add two-carbons to the growing chain, from a biochemical and physiological stand-point, increasing chain length and secreting/transporting the products to the epicuticle represents a facile mechanism to thwart the growth of potential pathogens. Successful pathogens, in turn must develop mechanisms for bypassing and/or degrading the ever-growing hydrocarbon chain lengths. In short, could fungal pathogens help explain the diversity and chain length of insect hydrocarbons on the one hand, and the evolution of specific enzymes (cytochrome P450s) and pathways to assimilate these lipids on the other? It should be noted that to date, there is little empirical evidence to support this idea, however, such a hypothesis does make certain predictions and can help explain certain phenomena. First, one would predict that insects that make longer chain hydrocarbons that *B. bassiana* cannot assimilate would be more resistant to fungal infection or vice versa that insects which display resistance to *B. bassiana* do so because of epicuticular lipid content, in particular by synthesizing longer and/or branched-chain hydrocarbons. Second, epicuticular hydrocarbon content may explain strain variation and sub-specificity seen between different fungal isolates. Finally, it is known that even amongst some (*B. bassiana*) susceptible insect species certain developmental stages (instars) are more resistant than others. Since epicuticular hydrocarbons are known to vary between such developmental stages, one could predict that these differences may help account for the variation in pathogen susceptibility observed.

## Concluding remarks

The lipid-rich insect epicuticle represents the first barrier against, and mediates the initial interaction with, microbial pathogens. Targeting of this layer by the entomopathogenic fungus *Beauveria bassiana* occurs within the context of a host-pathogen interaction. Aspects of the biochemical basis for fungal mediated hydrocarbon oxidation and assimilation have been uncovered. Genomic, genetic, and biochemical data indicate that the fungus contains a repertoire of cytochrome P450 enzymes, likely with over-lapping specificities, along with attendant downstream pathways, that act to assimilate insect hydrocarbons. There remain, however, many unanswered questions and significant aspects of the fungal-epicuticle interaction remains obscure. The substrate specificities of only one *B. bassiana* cytochrome P450 has been examined, and questions concerning the uptake and transport of hydrocarbons into the fungus have yet to be adequately addressed. The idea that epicuticular hydrocarbon synthesis and fungal assimilation of these compounds may represent a co-evolutionary race needs empirical support. Further research aimed toward examining these and other questions can yield novel insights into the biochemistry of hydrocarbon degradation as well as into the ecology and evolution of the interaction between fungal entomopathogens and their insect hosts, and can impact practical applications of fungi in biological control of insects and/or exploitation of the fungal hydrocarbon pathways in biotechnological applications.

## Materials and methods

### Chemical reagents and cultivation of fungi

*Beauveria bassiana* (ATCC 90517) was routinely grown on potato dextrose agar (PDA) or Czapek-Dox plates. Plates were incubated at 26°C for 10–15 days and aerial conidia were harvested by flooding the plate with sterile distilled water. Conidial suspensions were filtered through a single layer of Miracloth and final spore concentrations were determined by direct count using a hemocytometer. The *S. cereviseae* WAT11 strain was used for heterologous expression of *B. bassiana* cytochrome P450s. Yeast cells were grown at 30°C in Difco Yeast Nitrogen Base medium without amino acids (YNB-aa, 6.7 g/L) containing glucose or galactose at 2%(w/v) with supplements as indicated. Chemical reagents were obtained from either Fischer Scientific or Sigma-Aldrich chemicals unless otherwise noted. Phosphinothricin was purchased from Gold Biotech or purified in the lab from the herbicide Finale (AgrEvo, Montvale, NJ) as described (Metzenberg et al., 2000).

### Nucleic acid manipulations and construction of *B. bassiana* cytochrome P450 knockout strains

All primer sequences for the nucleic acid manipulations, RT-PCR, Southern blot probe generation, and yeast expression vector construction are listed in Table 3. To generate the vector for construction of the cytochrome P450 knockout strains in *B. bassiana* via homologous recombination, fragments for each gene (see Table 1 for list of P450s and accession numbers) were amplified from genomic DNA using the primer pairs as listed in Table 3. PCR products was cloned into the pCR2.1-TOPO blunt-end vector (Invitrogen) generating pTOPO-*Bbcyp450xxx* clones for each gene. Long-range deletion inverse PCR using the primer pair for each gene (Table 3) were then used to produce linear fragments lacking 50–200 bp of internal sequence for each gene using the respective pTOPO-*Bbcyp450* plasmid of each gene as template. The generated linear DNA was then blunt-end ligated to a PCR product corresponding to the herbicide resistance gene (*bar*) cassette amplified from pBAR-GPE (Sweigard et al., 1997) using primer pair pBARF and pBARR. The integrity of each resultant gene replacement plasmid designated as pKO-*Bbcyp450xxx*, was confirmed by PCR and sequencing. Preparation of competent cells, transformation, and screening of recombinant clones was performed as described using a PEG-LiAc mediated protocol (Zhang et al., 2010; Fan et al., 2011). The transformation mixture (0.25–0.5 ml) was plated onto Czapek-Dox medium containing 200 mg/ml phosphinothricin, 0.01% bromocresol purple, pH 6.3, in 150 mm diameter Petri dishes overlaid with a sheet of sterilized cellophane. Genomic DNA was isolated as described (Liu et al., 2000). Putative *B. bassiana* gene knockout clones were screened and verified by PCR analysis using primers designed to each *Bbcyp450* gene (Table 3). PCR reactions were performed using the following protocol: 95°C for 3 min, followed by 35 cycles of denaturation at 95°C for 30 s, annealing at 56°C for 30 s, and extension at 72°C for 1 min. Transformants were confirmed by PCR and RT-PCR using primers as listed in Table 3.

**Table 3:** **Primers used in this study**.

**Name**	**Sequence (5′ to 3′)**	**Use**
pBARF	GTCGACAGAAGATGATATTGAAGG	KO strain construction
pBARR	TCATCAGATCTCGGTGACGGGCAGG	KO strain construction
**CYP5337A1 KNOCKOUT**		
P4F	GTGTGCGTGATCCAGAGCTCTGC	KO strain construction
P4R	GCACCAAGTTTCGAGACTGGGACAT	KO strain construction
P4KOF	ACTTACGACCTATGCAGATGCGC	KO strain construction
P4KOR	GTTGGCTTGTATGGGATTACGCC	KO strain construction
P4RTPCRF	CACATTGTTGTACGCGGTACTTTGC	KO screening
P4RTPCRR	TATGGTCCGGATGCAATGGAGTGG	KO screening
**CYP52G11 KNOCKOUT**		
P5F	CGCTCACTGCTATCCTCATCGGC	KO strain construction
P5R	CAGAACAGCGATAACGTGACGAGCT	KO strain construction
P5KOF	CAAGGGCGGCTGGGAATATCTC	KO strain construction
P5KOR	TTCTCGTAGCCGCCAAAGGTCT	KO strain construction
P5RTPCRF	CTCCTCAACGTCCTCCTCGCCGG	KO screening
P5RTPCRR	GAGGAGTCGGGCGAGGACGTAAC	KO screening
**CYP617N1 KNOCKOUT**		
P7F	GAAAGCCCCAACGAAGGCCTGAT	KO strain construction
P7R	GAAGTTGCCCGGTCCCTTGTCAA	KO strain construction
P7KOF	GGACAAGTCTCTTCTTGACGAAAGCA	KO strain construction
P7KOR	ATTGGTCGCATTCTTGCCGCC	KO strain construction
P7RTPCRF	TGGGATTGGTGCCGTGGGCAA	KO screening
P7RTPCRR	CCTGTATGCACATTTCCATTCCGCC	KO screening
**CYP53A26 KNOCKOUT**		
P8F	CCGTCATTGTCCCGAGCCAAGAA	KO strain construction
P8R	TGGCAACGGATGCAAAGACCAAGAC	KO strain construction
P8KOF	CTGGACGCCGTCATTCCCGAG	KO strain construction
P8KOR	CGGGTTCTCGATTCGGCTCTTG	KO strain construction
P8RTPCRF	ATTCGGCATGTTGGCGAGTGGTAT	KO screening
P8RTPCRR	AGGTCGGCACGCTCAAGACGGT	KO screening
**CYP655C1 KNOCKOUT**		
Ep(2)F	GCCTCACGCAACTACTCAGCCTTTCATC	KO strain construction
Ep(2)R	CGCAGACTCATTCTGGACCATCATTTG	KO strain construction
Ep(2)KOF	CAAACTATGGCGGCTACCGGT	KO strain construction
Ep(2)KOR	GTCGTGAGCTAGGAATCTGCGCA	KO strain construction
Ep(2)RTPCRF	CTCCTAGACAGCGTGAGCCTCCCAT	KO screening
Ep(2)RTPCRR	GGTGCTGCCTGATGGGCTCGAC	KO screening
**CYP5337A1 EXPRESSION**		
EP(4)F	GGATTAATAATGGCGCTCACTGCTATCCT	
EP(4)R	GGGTTAATTTAGTGGTGGTGGTGGTGG	
	TGGACAGCCTCGTGCAGGCGGAC	
**CYP617N1 EXPRESSION**		
EP(7)F	GGATTAATAATGGCCGTGGTTGAGCTC	
EP(7)R	GGGTTAATTTAGTGGTGGTGGTGGTGGT	
	GTCGCCTAGCCAGTGCATCTC	
**CYP53A26 EXPRESSION**		
EP(8)F	GGATTAATAATGGCTCTCGGCCAACTT	
	GCC	
EP(8)R	GGGTTAATTTAGTGGTGGTGGTGGTGGT	
	GTTGCAGCTTTTCTTCTGCATTCTG	
ActinF	TTGGTGCGAAACTTCAGCGTCTAGTC	RT-PCR
ActinR	TCCAGCAAATGTGGATCTCCAAGCAG	

### Growth and grasshopper wing germination assays

Fungal growth experiments of various hydrocarbons were performed as follows; fungal spores were harvested from PDA plates directly into sterile distilled water and were washed twice with the same solution; the suspension was then adjusted to 10^7^–10^8^ conidia ml^−1^ after counting using a hemocytometer. Spore suspensions (5–10 μl) were then placed into the middle of microtiter agar plates (24- or 48-well). For 24-well plates, each well contained 1 ml MM in Noble agar overlaid with the desired alkane (0.1 ml of a10% hydrocarbon stock solution in hexane) as a carbon source. Substrates tested included the following alkanes (0.1%); C_9_, C_10_, C_12_, C_14_, C_16_, C_18_, C_24_, and C_28_. Grasshopper wings sterilized using 37% H_2_O_2_ were immersed in a conidial suspension in water at a concentration of 1 × 10^6^ spores/ml for 20 s, and placed on 0.7% water agar. After incubation for 18 h, the germinated conidia were counted under a light microscope.

### Heterologous expression of *B. bassiana* cytochrome P450s in yeast

The coding region of CYP5337A1, CYP617N1, CYP53A26, and CYP584Q1 genes were cloned from a *B. bassiana* cDNA library by PCR using primer pairs as listed in Table 3. The resultant PCR product corresponding to each gene was designed to contain an 18 bp (6 amino acid) histidine tag and the products were cloned into pYeDP60 under the control of a GAL1 promoter. The sequence integrity of plasmid inserts were confirmed by sequencing and plasmids were then transformed into *S. cerevisiae* WAT11, a yeast strain engineered and optimized for cytochrome P450 expression, using a lithium acetate protocol (Pompon et al., 1996). Transformants were selected on YNB w/o amino acids, 2% glucose and auxotrophic supplements and the expression strain designated as *Sc*-*Bbcyp5337a1*, *Sc*-*Bbcyp617N1*, *Sc*-*Bbcyp53a26*, and *Sc*-*Bbcyp584q1*, respectively.

Yeast cultures were grown and heterologous expression of each CYP (CYP5337A1, CYP617N1, CYP53A26, and CYP584Q1) was induced as described in Pompon et al. (Pompon et al., 1996) from one isolated transformed colony. Briefly, after growth, cells were harvested by centrifugation and manually broken with glass beads (0.45 mm diameter) in 50 mM Tris-HCl buffer (pH 7.5) containing 1 mM EDTA and 600 mM sorbitol. The homogenate was centrifuged for 10 min at 10,000 g. The resulting supernatant was centrifuged for 1 h at 100,000 g. The pellet consisting of microsomal membranes was resuspended in 50 mMTris-HCl (pH 7.4), 1 mM EDTA and 30% (v/v) glycerol with a Potter-Elvehjem homogenizer and stored at −30°C. The volume of resuspension buffer is proportional to the weight of yeast pellet: microsomes extracted from 6 g of yeast are resuspended in 3 ml of buffer. All procedures for microsomal preparation were carried out at 0–4°C. Western blots were performed using standard protocols. Mouse anti-His monoclonal antibodies were obtained from Invitrogen.

### CYP solubilization and partial purification

Microsomal fractions were treated with 10% (w/v) sodium cholate solution (final concentration 1% w/v) for 30 min at 4°C, in order to solubilize proteins from membranes. After ultra-centrifugation (100,000 g, 60 min, 4°C) supernatants were loaded onto Ni-NTA columns and purified by immobilized metal-ion affinity chromatography (IMAC) following standards protocols. Eluted fractions were analyzed by SDS–PAGE. Fractions containing the tagged protein were pooled, concentrated, and assayed for P450 content.

### Measurement of reduced carbon monoxide (CO) difference spectra

The reduced CO difference spectra were measured in an Ultrospec 2100 pro spectrophotometer (Biochrom Ltd., Cambridge, UK) as described (Schenkman and Jansson, 2006), with minimal modifications. Briefly, samples were diluted to 1 mg ml^−1^ protein, CO was slowly burbled into the cuvet for about 30 s, and a few milligrams of sodium dithionite were added. After stirring and waiting 1 min, the difference spectrum was recorded between 400 and 500 nm. Protein concentrations were determined by the Pierce bicinchoninic acid microassay, using bovine serum albumin as standard.

### Conflict of interest statement

The authors declare that the research was conducted in the absence of any commercial or financial relationships that could be construed as a potential conflict of interest.

## References

[B1] Anderson S. O. Hojrup P. Roepstorff P. (1995). Insect cuticular proteins. Insect Biochem. Mol. Biol. 25, 153–176 10.1016/0965-1748(94)00052-J 7711748

[B2] Asiimwe T. Krause K. Schlunk I. Kothe E. (2012). Modulation of ethanol stress tolerance by aldehyde dehydrogenase in the mycorrhizal fungus *Tricholoma vaccinum*. Mycorrhiza 22, 1–14 10.1007/s00572-011-0424-9 22159964

[B3] Bagnères A. G. Blomquist G. J. (2010). Site of synthesis, mechanism of transport and selective deposition of hydrocarbons, in Insect Hydrocarbons: Biology, Biochemistry and Chemical Ecology, eds Blomquist G. J. Bagnères A. G. (New York, NY: Cambridge University Press), 75–99

[B4] Berbee M. L. Taylor J. W. (2001). Fungal molecular evolution: gene trees and geologic time, in The Mycota VII Part, B, eds McLaughlin McLaughlin Lemke (Berlin Heidelberg: Springer-Verlag), 229–245

[B5] Bidochka M. J. Clark D. C. Lewis M. W. Keyhani N. O. (2010). Could insect phagocytic avoidance by entomogenous fungi have evolved via selection against soil amoeboid predators? Microbiology 156, 2164–2171 10.1099/mic.0.038216-0 20338910

[B6] Bidochka M. J. Khachatourians G. G. (1991). The implication of metabolic acids produced by *Beauveria bassiana* in pathogenesis of the migratory grasshopper, *Melanoplus sanguinipes*. J. Invertebr. Pathol. 58, 106–117

[B7] Binnington K. Retnakaran A. (1991). Physiology of the Insect Epidermis. Melbourne, CSIRO Publications

[B8] Blomquist G. J. Dillwith J. W. (1985). Cuticular lipids, in Comprehensive Insect Physiology, Biochemistry, and Pharmacology, eds Kerkut G. A. Gilbert L. I. (Oxford: Pergamon Press), 117–154

[B9] Blomquist G. J. Nelson D. R. Derenobales M. (1987). Chemistry, biochemistry, and physiology of insect cuticular lipids. Arch. Insect Biochem. Physiol. 6, 227–265

[B10] Blomquist G. J. Tillman-Wall J. A. Guo L. Quilici D. R. Gu P. Schal C. (1993). Hydrocarbon and hydrocarbon-derived sex pheromones in insects, in Biochemistry and Endocrine Regulation, eds Stanley-Samuelson D. W. Nelson D. R. (Lincoln, NE: University of Nebraska Press), 317–351

[B11] Boucias D. G. Pendland J. C. (1984). Nutritional requirements for conidial germination of several host range pathotypes of the entomopathogenic fungus *Nomuraea rileyi*. J. Invertebr. Pathol. 43, 288–292

[B12] Boucias D. G. Pendland J. C. Latge J. P. (1988). Nonspecific factors involved in attachment of entomopathogenic Deuteromycetes to host insect cuticle. Appl. Environ. Microbiol. 54, 1795–1805 16347689 10.1128/aem.54.7.1795-1805.1988 PMC202748

[B13] Buckner J. S. (1993). Cuticular polar lipids of insects, in Insect Lipids: Chemistry, Biochemistry and Biology, eds Stanley-Samuelson D. W. Nelson D. R. (Lincoln, NE: University of Nebraska Press), 227–270

[B14] Cameotra S. S. Singh H. D. Hazarika A. K. Baruah J. N. (1983). Mode of uptake of insoluble solid substrates by microorganisms 2. Uptake of solid normal-alkanes by yeast and bacterial species. Biotechnol. Bioeng. 25, 2945–2956 10.1002/bit.260251211 18548629

[B15] Charnley A. K. St Leger R. (1991). The role of cuticle degrading enzymes in fungal pathogenesis of insects, in The Fungal Spore and Disease Initiation, eds Cole G. T. Hoch H. C. (New York, NY: Plenum Press), 267–286

[B16] Chino H. Kitazawa K. (1981). Diacylglycerol-carrying lipoprotein of hemolymph of the locust and some insects. J. Lipid Res. 22, 1042–1052 6795289

[B17] Cho E. M. Boucias D. Keyhani N. O. (2006a). EST analysis of cDNA libraries from the entomopathogenic fungus *Beauveria (Cordyceps) bassiana*. II. Fungal cells sporulating on chitin and producing oosporein. Microbiology 152, 2855–2864 10.1099/mic.0.28845-0 16946279

[B18] Cho E. M. Liu L. Farmerie W. Keyhani N. O. (2006b). EST analysis of cDNA libraries from the entomopathogenic fungus *Beauveria* (*Cordyceps*) *bassiana*. I. Evidence for stage-specific gene expression in aerial conidia, in vitro blastospores and submerged conidia. Microbiology 152, 2843–2854 10.1099/mic.0.28844-0 16946278

[B19] Clarkson J. M. Charnley A. K. (1996). New insights into the mechanisms of fungal pathogenesis in insects. Trends Microbiol. 4, 197–203 10.1016/0966-842X(96)10022-6 8727600

[B20] Cooney J. J. Siporin C. Smucker R. A. (1980). Physiological and cytological responses to hyd rocarbons by the hydrocarbon-using fungus *Cladosporium resinae*. Botanica Marina 23, 227–232

[B21] Crespo R. Juarez M. P. Cafferata L. F. R. (2000). Biochemical interaction between entomopathogenous fungi and their insect-host-like hydrocarbons. Mycologia 92, 528–536

[B22] Crespo R. Juarez M. P. Dal Bello G. M. Padin S. Fernandez G. C. Pedrini N. (2002). Increased mortality of *Acanthoscelides obtectus* by alkane-grown *Beauveria bassiana*. Biocontrol 47, 685–696

[B23] Crespo R. Pedrini N. Juarez M. P. Dal Bello G. M. (2008). Volatile organic compounds released by the entomopathogenic fungus *Beauveria bassiana*. Microbiol. Res. 163, 148–151 10.1016/j.micres.2006.03.013 16733086

[B24] Deng J. Carbone I. Dean R. A. (2007). The evolutionary history of Cytochrome P450 genes in four filamentous Ascomycetes. BMC Evol. Biol. 7:30 10.1186/1471-2148-7-30 17324274 PMC1828051

[B25] Dereeper A. Audic S. Claverie J.-M. Blanc G. (2010). BLAST-EXPLORER helps you building datasets for phylogenetic analysis. BMC Evol. Biol. 10:8 10.1186/1471-2148-10-8 20067610 PMC2821324

[B26] Dereeper A. Guignon V. Blanc G. Audic S. Buffet S. Chevenet F. (2008). Phylogeny.fr: robust phylogenetic analysis for the non-specialist. Nucleic Acids Res. 36, 465–469 10.1093/nar/gkn180 18424797 PMC2447785

[B27] Dettner K. Liepert C. (1994). Chemical mimicry and camouflage. Annu. Rev. Entomol. 39, 129–154.

[B28] Doddapaneni H. Chakraborty R. Yadav J. S. (2005). Genome-wide structural and evolutionary analysis of the P450 monooxygenase genes (P450ome) in the white rot fungus Phanerochaete chrysosporium: evidence for gene duplications and extensive gene clustering. BMC Genomics 6:92 10.1186/1471-2164-6-92 15955240 PMC1184071

[B29] Espelie K. Chapman R. F. Sword G. A. (1994). Variation in the surface lipids of the grasshopper, *Schistocerca americana* (Drury). Biochem. Syst. Ecol. 22, 563–575

[B30] Fan Y. Borovsky D. Hawkings C. Ortiz-Urquiza A. Keyhani N. O. (2012a). Exploiting host molecules to augment mycoinsecticide virulence. Nat. Biotechnol. 30, 35–37 10.1038/nbt.2080 22231090

[B31] Fan Y. Pereira R. M. Kilic E. Casella G. Keyhani N. O. (2012b). Pyrokinin beta-neuropeptide affects necrophoretic behavior in fire ants (*S. invicta*), and expression of beta-NP in a mycoinsecticide increases its virulence. PLoS ONE 7:e26924 10.1371/journal.pone.0026924 22238569 PMC3251551

[B32] Fan Y. Zhang S. Kruer N. Keyhani N. O. (2011). High-throughput insertion mutagenesis and functional screening in the entomopathogenic fungus *Beauveria bassiana*. J. Invertebr. Pathol. 106, 274–279 10.1016/j.jip.2010.11.003 21059351

[B33] Ferron P. (1981). Pest control by the fungi Beauveria and Metarhizium, in Microbial Control of Pests and Plant Diseases 1970-1980, ed Burges H. D. (New York, NY: Academic Press), 465–482

[B34] Fickers P. Benetti P. H. Wache Y. Marty A. Mauersberger S. Smit M. S. (2005). Hydrophobic substrate utilisation by the yeast *Yarrowia lipolytica*, and its potential applications. Fems Yeast Res. 5, 527–543 10.1016/j.femsyr.2004.09.004 15780653

[B35] Flipphi M. Mathieu M. Cirpus I. Panozzo C. Felenbok B. (2001). Regulation of the aldehyde dehydrogenase gene (aldA) and its role in the control of the coinducer level necessary for induction of the ethanol utilization pathway in *Aspergillus nidulans*. J. Biol. Chem. 276, 6950–6958 10.1074/jbc.M005769200 11102439

[B36] Freimoser F. M. Hu G. St. Leger R. J. (2005). Variation in gene expression patterns as the insect pathogen *Metarhizium anisopliae* adapts to different host cuticles or nutrient deprivation *in vitro*. Microbiology 151, 361–371 10.1099/mic.0.27560-0 15699187

[B37] Gao Q. Jin K. Ying S.-H. Zhang Y. Xiao G. Shang Y. (2011). Genome sequencing and comparative transcriptomics of the model entomopathogenic fungi *Metarhizium anisopliae* and *M. acridum*. PLoS Genetics 7:e1001264. 10.1371/journal.pgen.1001264 21253567 PMC3017113

[B38] Gartner C. A. Thompson S. J. Rettie A. E. Nelson S. D. (2001). Human aromatase in high yield and purity by perfusion chromatography and its characterization by difference spectroscopy and mass spectrometry. Protein Expr. Purif. 22, 443–454 10.1006/prep.2001.1464 11483007

[B39] Gibbs A. Mousseau T. A. (1994). Thermal acclimation and genetic variation in cuticular lipids of the lesser migratory grasshopper (*Melanoplus sanguinipes*) - effects of lipid composition on biophysical properties. Physiol. Zool. 67, 1523–1543

[B40] Gibbs A. Mousseau T. A. Dingle H. Crowe J. H. (1990). Genetic and acclimatory variation in cuticle lipids of grasshoppers, *Melanoplus sanguinipes*. Am. Zool. 30, A33–A33

[B41] Goettel M. S. Inglis G. D. Wraight S. P. (2000). Fungi, in Field Manual of Techniques in Invertebrate Pathology, eds Lacey L. A. Kaya H. K. (Netherlands: Kluwer Academic), 255–282

[B42] Golebiowski M. Malinski E. Bogus M. I. Kumirska J. Stepnowski P. (2008). The cuticular fatty acids of *Calliphora vicina*, *Dendrolimus pini* and *Galleria mellonella* larvae and their role in resistance to fungal infection. Insect Biochem. Mol. Biol. 38, 619–627 10.1016/j.ibmb.2008.03.005 18510973

[B43] Goma G. Pareilleux A. Durand G. (1973). Cinetique de degradation des hydrocarbures par *Candida lipolytica*. Arch. Mikrobiol. 88, 97–109 4684078

[B44] Gu X. Quilici D. Juarez P. Blomquist G. J. Schal C. (1995). Biosynthesis of hydrocarbons and contact sex pheromone and their transport by lipophorin in females of the German cockroach (*Blattella germanica*). J. Insect Physiol. 41, 257–267.

[B45] Hackman R. H. (1984). Cuticle: biochemistry, in Biology of the Integument, eds Bereiter-Hahn J. Mateltsy A. G. Richards K. S. (Berlin: Springer-Verlag), 583–610

[B46] Harada N. (1998). Novel properties of human placental aromatase as cytochrome P450. Purification and characterization of a unique form of aromatase. J. Biochem. 103, 106–113 3129418 10.1093/oxfordjournals.jbchem.a122213

[B47] Holder D. J. Keyhani N. O. (2005). Adhesion of the entomopathogenic fungus *Beauveria (Cordyceps) bassiana* to substrata. Appl. Environ. Microbiol. 71, 5260–5266 10.1128/AEM.71.9.5260-5266.2005 16151112 PMC1214598

[B48] Howard R. W. (1993). Cuticular hydrocarbons and chemical communication, in Insect Lipids: Chemistry, Biochemistry and Biology, eds Stanley-Samuelson D. R. Nelson D. R. (Lincoln, NE: University of Nebraska Press), 179–226

[B49] Howard R. W. Blomquist G. J. (2005). Ecological, behavioral, and biochemical aspects of insect hydrocarbons. Annu. Rev. Entomol. 50, 371–393 10.1146/annurev.ento.50.071803.130359 15355247

[B50] Hung C. Y. Boucias D. G. (1992). Influence of *Beauveria bassiana* on the cellular defense response of the *Beet Armyworm*, *Spodoptera exigua*. J. Invertebr. Pathol. 60, 152–158 10.1006/jipa.1993.1032 8463710

[B51] Inglis G. D. Goettel M. S. Butt T. M. Strasser H. (2001). Use of hyphomycetous fungi for managing insect pests, in Field Manual of Techniques in Invertebrate Pathology, eds Lacey L. A. Kaya H. K. (Netherlands: Kluwer Academic), 651–679

[B52] Ito H. Fukuda Y. Murata K. Kimura A. (1983). Transformation of intact yeast cells treated with alkali cations. J. Bacterol. 153, 163–168 6336730 10.1128/jb.153.1.163-168.1983 PMC217353

[B53] Jarrold S. L. Moore D. Potter U. Charnley A. K. (2007). The contribution of surface waxes to pre-penetration growth of an entomopathogenic fungus on host cuticle. Mycol. Res. 111, 240–249 10.1016/j.mycres.2006.10.007 17324760

[B54] Juárez M. P. Pedrini N. Crespo R. (2004). Mycoinsecticides against Chagas disease vectors: biochemistry involved in insect host hydrocarbon degradation, in Multidisciplinarity for Parasites, Vectors and Parasitic Diseases, ed Mas-Comas S. (Bologna: Monduzzi Editore), 137–142

[B55] Kappeli O. Walther P. Mueller M. Fiechter A. (1984). Structure of the cell-surface of the yeast *Candida tropicalis* and its relation to hydrocarbon transport. Arch. Microbiol. 138, 279–282 6477032 10.1007/BF00410890

[B56] Kerwin J. L. (1984). Fatty acid regulation of the germination of *Erynia variabilis* conidia on adults and puparia of the lesser housefly, *Fannia canicularis*. Can. J. Microbiol. 30, 158–161

[B57] Khachatourians G. G. (1996). Biochemistry and Molecular Biology of Entomopathogenic Fungi, in The Mycota VI: Human and Animal Relationships, eds Howard D. H. Miller J. D. (Berlin, Heidelberg: Springer-Verlag), 331–363

[B58] Kirkland B. H. Cho E. M. Keyhani N. O. (2004a). Differential susceptibility of *Amblyomma maculatum* and *Amblyomma americanum* (Acari: Ixodidea) to the entomopathogenic fungi *Beauveria bassiana* and *Metarhizium anisopliae*. Biol. Control 31, 414–421

[B59] Kirkland B. H. Westwood G. S. Keyhani N. O. (2004b). Pathogenicity of entomopathogenic fungi *Beauveria bassiana* and *Metarhizium anisopliae* to Ixodidae tick species *Dermacentor variabilis*, *Rhipicephalus sanguineus*, and *Ixodes scapularis*. J. Med. Entomol. 41, 705–711 15311464 10.1603/0022-2585-41.4.705

[B60] Kirkland B. H. Eisa A. Keyhani N. O. (2005). Oxalic acid as a fungal acaracidal virulence factor. J. Med. Entomol. 42, 346–351 15962786 10.1093/jmedent/42.3.346

[B61] Koidsumi K. (1957). Antifungal action of cuticular lipids in insects. J. Insect Physiol. 1, 40–51

[B62] Kurtti T. J. Keyhani N. O. (2008). Intracellular infection of tick cell lines by the entomopathogenic fungus *Metarhizium anisopliae*. Microbiology 154, 1700–1709 10.1099/mic.0.2008/016667-0 18524924

[B63] Latge J. P. Sampedro L. Brey P. Diaquin M. (1987). Aggressiveness of *Conidiobolus obscurus* against the pea aphid - influence of cuticular extracts on ballistospore germination of aggressive and nonaggressive Strains. J. Gen. Microbiol. 133, 1987–1997

[B64] Lau S. M. Harder P. A. O'Keefe D. P. (1993). Low carbon monoxide affinity allene oxide synthase is the predominant cytochrome P450 in many plant tissues. Biochemistry 32, 1945–1950 8448153 10.1021/bi00059a010

[B65] Lecuona R. Clement J. L. Riba G. Joulie C. Juarez P. (1997). Spore germination and hyphal growth of *Beauveria* sp on insect lipids. J. Econ. Entomol. 90, 119–123

[B66] Lecuona R. Riba G. Cassier P. Clement J. L. (1991). Alterations of insect epicuticular hydrocarbons during infection with *Beauveria bassiana* or *B*. brongniartii. J. Invertebr. Pathol. 58, 10–18

[B67] Lewis M. W. Robalino I. V. Keyhani N. O. (2009). Uptake of the fluorescent probe FM4-64 by hyphae and haemolymph-derived *in vivo* hyphal bodies of the entomopathogenic fungus *Beauveria bassiana*. Microbiology 155, 3110–3120 10.1099/mic.0.029165-0 19542008

[B68] Lindley N. D. (1995). Bioconversion and biodegradation of aliphatic-hydrocarbons. Can. J. Bot. 73, S1034–S1042

[B69] Lindley N. D. Heydeman M. T. (1986). The uptake of normal-alkanes from alkane mixtures during growth of the hydrocarbon-utilizing fungus *Cladosporium resinae*. Appl. Microbiol. Biotechnol. 23, 384–388.

[B70] Liu D. Coloe S. Baird R. Pedersen J. (2000). Rapid mini-preparation of fungal DNA for PCR. J. Clin. Microbiol. 38, 471–471 10681211 10.1128/jcm.38.1.471-471.2000 PMC88759

[B71] Lockey K. H. (1988). Lipids of the insect cuticle - origin, composition and function. Comp. Biochem. Physiol. B Biochem. Mol. Biol. 89, 595–645

[B72] Lockey K. H. Oraha V. S. (1990). Cuticular lipids of adult *Locusta migratoria* migratoriodes (R and F), *Schistocerca gregaria* (Forskal) (Acrididae) and Other Orthopteran Species.2. Hydrocarbons. Comp. Biochem. Physiol. B Biochem. Mol. Biol. 95, 721–744

[B73] Lord J. C. Howard R. W. (2004). A proposed role for the cuticular fatty amides of *Liposcelis bostrychophila* (Psocoptera: Liposcelidae) in preventing adhesion of entomopathogenic fungi with dry-conidia. Mycopathologia 158, 211–217 15518350 10.1023/b:myco.0000041837.29478.78

[B74] Marchler-Bauer A. Lu S. Anderson J. B. Chitsaz F. Derbyshire M. K. Deweese-Scott C. (2011). CDD: a conserved domain database for the functional annotation of proteins. Nucleic Acids Res. 39, D225–D229 10.1093/nar/gkq1189 21109532 PMC3013737

[B75] Meisel M. N. Medvedeva G. A. Kozlova T. M. Domoshnikova N. A. Zaikina A. I. Fedoseeva G. E. (1973). Regularities of penetration into yeast cells of higher fatty acids and hydrocarbons, their intracellular migration and concentration, in Proceedings of the 3rd International Specilized Symposium on Yeast, eds Suomalainen H. Waller C. (Helsinki: Otaniemi), 149–168

[B76] Metzenberg R. L. Jacobson D. J. Bertrand H. (2000). Making the selective agent for the *Bar* plasmids, phosphinothricin (glufosinate) affordable for routine use. Fungal Genet. Newsl. 47, 79–80

[B77] Montellano P. R. O. D. (ed.). (2005). Cytochrome P450: Structure, Mechanism, and Biochemistry. New York, NY: Kluwer Academic/Plenum

[B78] Napolitano R. Juarez M. P. (1997). Entomopathogenous fungi degrade epicuticular hydrocarbons of *Triatoma infestans*. Arch. Biochem. Biophys. 344, 208–214 10.1006/abbi.1997.0163 9244399

[B79] Nelson D. R. (1993). Methyl-branched lipids in insects, in Insect lipids: Chemistry, Biochemistry and Biolgy, eds Stanley-Samuelson D. W. Nelson D. R. (Lincoln, NE: University of Nebraska Press), 271–315

[B80a] Nelson D. R. Koymans L. Kamataki T. Stegeman J. J. Feyereisen R. Waxman D. J. (1996). P450 superfamily: update on new sequences, gene mapping, accession numbers and nomenclature. Pharmacogenetics 6, 1–42 8845856 10.1097/00008571-199602000-00002

[B80] Nelson D. R. (1998). Metazoan cytochrome P450 evolution. Comp. Biochem. Physiol. C Pharmacol. Toxicol. Endocrinol. 121, 15–22 9972448 10.1016/s0742-8413(98)10027-0

[B81] Nelson D. R. (1999). Cytochrome P450 and the individuality of species. Arch. Biochem. Biophys. 369, 1–10 10.1006/abbi.1999.1352 10462435

[B82] Nelson D. R. (2009). The cytochrome P450 homepage. Hum. Genomics 4, 59–65 19951895 10.1186/1479-7364-4-1-59 PMC3500189

[B83] Nelson D. R. Blomquist G. J. (1995). Insect Waxes, in Waxes: Chemistry, Molecular Biology, and Functions, ed Hamilton R. J. (Dundee: The Oily Press, Ltd.), 1–90

[B84] Noble-Nesbitt J. I. E. (1991). Cuticular permeability and its control, in Physiology of the Insect Epidermis, eds Binnington K. Retnakaran A. (Melbourne: CSIRO), 252–283

[B85] Padilla-Guerrero I. E. Barelli L. Gonzalez-Hernandez G. A. Torres-Guzman J. C. Bidochka M. J. (2011). Flexible metabolism in *Metarhizium anisopliae* and *Beauveria bassiana*: role of the glyoxylate cycle during insect pathogenesis. Microbiology 157, 199–208 10.1099/mic.0.042697-0 20929953

[B86] Pedrini N. Crespo R. Juarez M. P. (2007). Biochemistry of insect epicuticle degradation by entomopathogenic fungi. Comp. Biochem. Physiol. C Toxicol. Pharmacol. 146, 124–137 10.1016/j.cbpc.2006.08.003 17052960

[B87] Pedrini N. Juárez M. Crespo R. De Alaniz M. (2006). Clues on the role of *Beauveria bassiana* catalases in alkane degradation events. Mycologia 98, 528–534 10.3852/mycologia.98.4.528 17144022

[B88] Pedrini N. Mijailovsky S. J. Girotti J. R. Stariolo R. Cardozo R. M. Gentile A. (2009). Control of pyrethroid-resistant chagas disease vectors with entomopathogenic fungi. PLoS Negl. Trop. Dis. 3:e434 10.1371/journal.pntd.0000434 19434231 PMC2674565

[B89] Pedrini N. Zhang S. Juárez M. Keyhani N. O. (2010). Molecular characterization and expression analysis of a suite of cytochrome P450 enzymes implicated in insect hydrocarbon degradation in the entomopathogenic fungus *Beauveria bassiana*. Microbiology 156, 2549–2557 10.1099/mic.0.039735-0 20413550

[B90] Pendland J. C. Hung S. Y. Boucias D. G. (1993). Evasion of host defense by *in vivo*-produced protoplast-like cells of the insect mycopathogen *Beauveria bassiana*. J. Bacteriol. 175, 5962–5969 8376342 10.1128/jb.175.18.5962-5969.1993 PMC206677

[B91] Pompon D. Louerat B. Bronine A. Urban P. (1996). Yeast expression of animal and plant P450s in optimized redox environments. Cytochrome P450(Pt B 272), 51–64 10.1016/S0076-6879(96)72008-6 8791762

[B92] Qiu Y. Tittiger C. Wicker-Thomas C. Le Goff G. Young S. Wajnberg E. (2012). An insect-specific P450 oxidative decarbonylase for cuticular hydrocarbon biosynthesis. Proc. Natl. Acad. Sci. U.S.A. 109, 14858–14863 10.1073/pnas.1208650109 22927409 PMC3443174

[B93] Reddy P. Singh H. Roy P. Baruah J. (1982). Predominant role of hydrocarbon solubilization in the microbial uptake of hydrocarbons. Biotechnol. Bioeng. 24, 1241–1269 10.1002/bit.260240603 18546423

[B94] Renobales M. Nelson D. R. Blomquist G. J. (1991). Cuticular Lipids, in Physiology of the Insect Epidermis, eds Binnington K. Retnakaran A. (Melbourne: CSIRO Publications), 240–251

[B95] Riley P. A. (1997). Melanin. In.t J. Biochem. Cell Biol. 29, 1235–1239 10.1016/S1357-2725(97)00013-7 9451820

[B96] Roberts D. W. Humber R. A. (1981). Entomogenous Fungi, in Biology of Conidial Fungi, eds Cole G. T. Kendrick B. (New York, NY: Academic Press), 201–236

[B97] Rojo F. (2010). Enzymes for aerobic degradation of alkanes, in Handbook of Hydrocarbon and Lipid Microbiology, ed Timmis K. N. (Berlin Heidelberg: Springer-Verlag), 781–797

[B98] Saito T. Aoki J. (1983). Toxicity of free fatty acids on the larval surfaces of 2 Lepidopterous insects towards *Beauveria bassiana* (Bals) Vuill and *Paecilomyces fumoso-roseus* (Wize) Brown Et Smith (Deuteromycetes, Moniliales). Appl. Entomol. Zool. 18, 225–233

[B99] Schal C. Sevala V. L. Young H. P. Bachmann J. A. S. (1998). Sites of synthesis and transport pathways of insect hydrocarbons: cuticle and ovary as target tissues. Am. Zool. 38, 382–393

[B100] Scheller U. Zimmer T. Becher D. Schauer F. Schunck W. H. (1998). Oxygenation cascade in conversion of n-alkanes to alpha, omega-dioic acids catalyzed by cytochrome p450 52A3. J. Biol. Chem. 273, 32528–32534 10.1074/jbc.273.49.32528 9829987

[B101] Schenkman J. B. Jansson I. (2006). Spectral analyses of cytochromes P450, in Methods in Molecular Biology: Cytochrome P450 Protocols, eds Phillips I. R. Shepard E. A. (New Jersey, NJ: Humana Press), 11–18 10.1385/1-59259-998-2:11 16719370

[B102] Scholte E. J. Ng'habi K. Kihonda J. Takken W. Paaijmans K. Abdulla S. (2005). An entomopathogenic fungus for control of adult African malaria mosquitoes. Science 308, 1641–1642 10.1126/science.1108639 15947190

[B103] Singer T. L. (1998). Roles of hydrocarbons in the recognition systems of insects. Am. Zool. 38, 394–405

[B104] Singh H. (2006). Fungal metabolism of petroleum hydrocarbons, in Mycoremediation: Fungal Bioremediation (Hoboken, NJ: John Wiley and Sons), 115–148

[B105] Smith R. J. Grula E. A. (1982). Toxic components on the larval surface of the Corn-Earworm (*Heliothis zea*) and their effects on germination and growth of *Beauveria bassiana*. J. Invertebr. Pathol. 39, 15–22

[B106] Steinhaus E. A. (1956). Microbial control-The emergence of an idea: a brief history of insect pathology through the nineteenth century. Hilgardia 26, 107–160

[B107] St Leger R. (1991). Integument as a Barrier to Microbial Infections, in Physiology of the Insect Epidermis, eds Binnington K. Retnakaran A. (Melbourne: CSIRO Publications), 284–306

[B108] Sweigard J. Chumley F. Carroll A. Farrall L. Valent B. (1997). A series of vectors for fungal transformation. Fungal Genet. Newsl. 44, 52–53

[B109] Takai H. Iwama R. Kobayashi S. Horiuchi H. Fukuda R. Ohta A. (2012). Construction and characterization of a *Yarrowia lipolytica* mutant lacking genes encoding cytochromes P450 subfamily 52. Fungal Genet. Biol. 49, 58–64 10.1016/j.fgb.2011.11.003 22119766

[B110] Van Beilen J. B. Li Z. Duetz W. A. Smits T. H. M. Witholt B. (2003). Diversity of alkane hydroxylase systems in the environment. Oil Gas Sci. Technol. 58, 427–440

[B111] Van Heusden M. C. Van Der Horst D. J. Ka-Wooya J. K. Law J. H. (1991). *In vivo* and *in vitro* loading of lipid by artificially lipid-depleted lipophorins: evidence for the role of lipophorin as a reusable lipid shuttle. J. Lipid Res. 32, 1789–1794 1770298

[B112] Van Valen L. (1973). A new evolutionary law. Evol. Theory 1, 1–30

[B113] Wanchoo A. Lewis M. W. Keyhani N. O. (2009). Lectin mapping reveals stage-specific display of surface carbohydrates in *in vitro* and haemolymph-derived cells of the entomopathogenic fungus *Beauveria bassiana*. Microbiology 155, 3121–3133 10.1099/mic.0.029157-0 19608611

[B114] Wang Z.-L. Zhang L.-B. Ying S.-H. Feng M.-G. (2012). Catalases play differentiated roles in the adaptation of a fungal entomopathogen to environmental stresses. Environ. Microbiol. 15, 409–418 10.1111/j.1462-2920.2012.02848.x 22891860

[B115] Xiao G. Ying S.-H. Zheng P. Wang Z.-L. Zhang S. Xie X.-Q. (2012). Genomic perspectives on the evolution of fungal entomopathogenicity in *Beauveria bassiana*. Sci. Rep. 2, 483 10.1038/srep00483 22761991 PMC3387728

[B116] Yadav J. S. Doddapaneni H. Subramanian V. (2006). P450ome of the white rot fungus Phanerochaete chrysosporium: structure, evolution and regulation of expression of genomic P450 clusters. Biochem. Soc. Trans. 34, 1165–1169 10.1042/BST0341165 17073777

[B117] Zhang S. Fan Y. Xia Y. X. Keyhani N. O. (2010). Sulfonylurea resistance as a new selectable marker for the entomopathogenic fungus *Beauveria bassiana*. Appl. Microbiol. Biotechnol. 87, 1151–1156 10.1007/s00253-010-2636-x 20449738

[B118] Zhang S. Widemann E. Bernard G. Lesot A. Pinot F. Pedrini N. (2012). CYP52X1, representing new cytochrome P450 subfamily, displays fatty acid hydroxylase activity and contributes to virulence and growth on insect cuticular substrates in entomopathogenicfungus *Beauveria bassiana*. J. Biol. Chem. 287, 13477–13486 10.1074/jbc.M111.338947 22393051 PMC3339963

